# Effect of Weight Loss via Severe vs Moderate Energy Restriction on Lean Mass and Body Composition Among Postmenopausal Women With Obesity: The TEMPO Diet Randomized Clinical Trial

**DOI:** 10.1001/jamanetworkopen.2019.13733

**Published:** 2019-10-30

**Authors:** Radhika V. Seimon, Anthony L. Wild-Taylor, Shelley E. Keating, Sally McClintock, Claudia Harper, Alice A. Gibson, Nathan A. Johnson, Hamish A. Fernando, Tania P. Markovic, Jacqueline R. Center, Janet Franklin, Peter Y. Liu, Stuart M. Grieve, Jim Lagopoulos, Ian D. Caterson, Nuala M. Byrne, Amanda Sainsbury

**Affiliations:** 1 The Boden Collaboration for Obesity, Nutrition, Exercise, and Eating Disorders, Faculty of Medicine and Health, Charles Perkins Centre, The University of Sydney, Camperdown, New South Wales, Australia; 2 School of Human Movement and Nutrition Sciences, Centre for Research on Exercise, Physical Activity and Health, The University of Queensland, Brisbane, Queensland, Australia; 3 Faculty of Health Sciences, The University of Sydney, Lidcombe, New South Wales, Australia; 4 Metabolism and Obesity Services, Royal Prince Alfred Hospital, Camperdown, New South Wales, Australia; 5 Bone Biology Program, Garvan Institute of Medical Research, St Vincent’s Hospital Clinical School, University of New South Wales, Sydney, New South Wales, Australia; 6 Division of Endocrinology, Department of Medicine, Harbor-University of California Los Angeles Medical Center and Los Angeles BioMedical Research Institute, Los Angeles; 7 Sydney Translational Imaging Laboratory, Heart Research Institute, Charles Perkins Centre, The University of Sydney, Camperdown, New South Wales, Australia; 8 Department of Radiology, Royal Prince Alfred Hospital, Camperdown, New South Wales, Australia; 9 Sunshine Coast Mind and Neuroscience–Thompson Institute, University of the Sunshine Coast, Queensland, Australia; 10 School of Health Sciences, College of Health and Medicine, University of Tasmania, Launceston, Tasmania, Australia

## Abstract

**Importance:**

Severely energy-restricted diets are the most effective dietary obesity treatment. However, there are concerns regarding potential adverse effects on body composition.

**Objective:**

To compare the long-term effects of weight loss via severe vs moderate energy restriction on lean mass and other aspects of body composition.

**Design, Setting, and Participants:**

The Type of Energy Manipulation for Promoting Optimum Metabolic Health and Body Composition in Obesity (TEMPO) Diet Trial was a 12-month, single-center, randomized clinical trial. A total of 101 postmenopausal women, aged 45 to 65 years with body mass index (calculated as weight in kilograms divided by height in meters squared) from 30 to 40, who were at least 5 years after menopause, had fewer than 3 hours of structured physical activity per week, and lived in the Sydney metropolitan area of New South Wales, Australia, were recruited between March 2013 and July 2016. Data analysis was conducted between October 2018 and August 2019.

**Intervention:**

Participants were randomized to either 12 months of moderate (25%-35%) energy restriction with a food-based diet (moderate intervention) or 4 months of severe (65%-75%) energy restriction with a total meal replacement diet followed by moderate energy restriction for an additional 8 months (severe intervention). Both interventions had a prescribed protein intake of 1.0 g/kg of actual body weight per day, and physical activity was encouraged but not supervised.

**Main Outcomes and Measures:**

The primary outcome was whole-body lean mass at 12 months after commencement of intervention. Secondary outcomes were body weight, thigh muscle area and muscle function (strength), bone mineral density, and fat mass and distribution, measured at 0, 4, 6, and 12 months.

**Results:**

A total of 101 postmenopausal women were recruited (mean [SD] age, 58.0 [4.2] years; mean [SD] weight, 90.8 [9.1] kg; mean [SD] body mass index, 34.4 [2.5]). Compared with the moderate group at 12 months, the severe group lost more weight (effect size, −6.6 kg; 95% CI, −8.2 to −5.1 kg), lost more whole-body lean mass (effect size, −1.2 kg; 95% CI, −2.0 to −0.4 kg), and lost more thigh muscle area (effect size, −4.2 cm^2^; 95% CI, −6.5 to −1.9 cm^2^). However, decreases in whole-body lean mass and thigh muscle area were proportional to total weight loss, and there was no difference in muscle (handgrip) strength between groups. Total hip bone mineral density (effect size, −0.017 g/cm^2^; 95% CI, −0.029 to −0.005 g/cm^2^), whole-body fat mass (effect size, −5.5 kg; 95% CI, −7.1 to −3.9 kg), abdominal subcutaneous adipose tissue (effect size, −1890 cm^3^; 95% CI, −2560 to −1219 cm^3^), and visceral adipose tissue (effect size, −1389 cm^3^; 95% CI, −1748 to −1030 cm^3^) loss were also greater for the severe group than for the moderate group at 12 months.

**Conclusions and Relevance:**

Severe energy restriction had no greater adverse effect on relative whole-body lean mass or handgrip strength compared with moderate energy restriction and was associated with 2-fold greater weight and fat loss over 12 months. However, there was significantly greater loss of total hip bone mineral density with severe vs moderate energy restriction. Therefore, caution is necessary when implementing severe energy restriction in postmenopausal women, particularly those with osteopenia or osteoporosis.

**Trial Registration:**

anzctr.org.au Identifier: 12612000651886

## Key Points

**Question:**

What are the long-term effects of severe vs moderate energy restriction on lean mass and other aspects of body composition?

**Findings:**

This randomized clinical trial included 101 postmenopausal women with obesity. At 12 months, participants who had undergone severe energy restriction experienced approximately 2-fold greater weight and fat loss, approximately 1.5 times as much loss of whole-body lean mass (proportional to total weight lost), and approximately 2.5 times as much loss of total hip bone mineral density compared with participants who had undergone moderate energy restriction.

**Meaning:**

Although severe energy restriction is an effective obesity treatment, caution is necessary when implementing it in postmenopausal women, especially those with osteopenia or osteoporosis.

## Teaser

This randomized clinical trial compares the long-term effects of weight loss via severe vs moderate energy restriction on lean mass and other aspects of body composition among postmenopausal women with obesity.

## Introduction

Effective obesity treatments are needed to reduce obesity-related morbidities and costs.^1,2^ The most effective dietary obesity treatments are severely energy-restricted diets^3,4^ of less than 800 kcal/d (<3350 kJ/d),^5^ which often involve replacing all or almost all foods with nutritionally replete meal replacement products, such as shakes, soups, or bars (ie, total diet replacement). Severely energy-restricted diets result in significantly greater short- and long-term^3,4,6,7,8^ weight loss compared with food-based diets that involve moderately restricting dietary energy intake by approximately 500 kcal/d (2100 kJ/d). In addition, total meal replacement diets are cheaper than the average per capita food expenditure in Australia and cost 3 times less to administer than food-based diets in terms of dietetics support.^9^

Despite being an effective and affordable dietary obesity treatment, a number of prominent clinical obesity treatment guidelines from around the world show limited support for the use of total meal replacement diets,^10,11^ and these diets are not routinely used by health care professionals.^9,12^ This may be because of reported adverse effects (eg, hair loss, constipation, headaches, dizziness, fatigue, and cholelithiasis),^6^ the lack of training and resources available for pretreatment evaluation and monitoring during these diets, and possibly also concerns that severe energy restriction may adversely affect body composition (ie, lean mass and bone mineral density [BMD]) compared with moderate energy restriction. For example, some studies have reported that larger energy deficits induce greater loss of fat-free mass in adults with overweight or obesity.^13,14,15,16^ In contrast, other studies have shown no difference in lean body mass following severe or moderate energy restriction in participants with obesity.^17,18^ As another example, some studies in women with overweight or obesity suggested that weight loss of more than 14% of initial weight during 3 to 4 months resulted in significant bone loss,^19,20^ whereas moderate weight loss of approximately 5% to 7% of initial body weight over 6 months resulted in little^21^ or no^22^ bone loss. In contrast, the Comprehensive Assessment of Long-term Effects of Reducing Intake of Energy (CALERIE) trial showed that men and women with overweight on a severely energy-restricted diet (albeit less restricted than the previously described studies) showed no greater loss of whole-body or hip BMD than individuals on a moderately energy-restricted diet.^23^ However, the studies cited here were not randomized clinical trials, were pilot studies, were of limited duration (ie, 6 days to 6 months), or were not specifically designed to assess muscle and bone health.

Although randomized clinical trials have evaluated the effects of severe vs moderate energy restriction on long-term weight maintenance in adults with overweight and obesity,^24,25^ in our review of the literature, no study directly compared the long-term effects of severe vs moderate energy restriction (to achieve fast vs slow weight loss, respectively) on lean mass and other aspects of body composition in people with obesity. We aimed to address this in the Type of Energy Manipulation for Promoting Optimum Metabolic Health and Body Composition in Obesity (TEMPO) Diet Trial.

## Methods

### Study Design and Participants

The TEMPO Diet Trial was a single-center, randomized clinical trial. Ethical approval was obtained from the Sydney Local Health District, Royal Prince Alfred Hospital Human Research Ethics Committee. It was conducted at the Charles Perkins Centre Royal Prince Alfred Clinic on the University of Sydney campus in Camperdown, New South Wales, Australia, with magnetic resonance imaging (MRI) and magnetic resonance spectroscopy (MRS) scans performed at I-Med Radiology (Camperdown). Reporting in this article is aligned with the Consolidated Standards of Reporting Trials (CONSORT) reporting guideline, and the full trial protocol can be found in Supplement 1.

Key inclusion criteria were postmenopausal women aged 45 to 65 years with body mass index (calculated as weight in kilograms divided by height in meters squared) from 30 to 40, at least 5 years after menopause, with less than 3 hours of structured physical activity per week (ie, sedentary), and living in the Sydney metropolitan area of New South Wales, Australia. We included women with this menopausal status for 2 reasons: first, to circumvent known effects of female hormone cycles and the menopausal transition on parameters under investigation in the trial and, second, because women older than 50 years have a 4-fold higher rate of osteoporosis and a 2-fold higher rate of osteopenia than men.^26^ Participants with osteoporosis or diabetes and those taking medication affecting body composition were excluded. The full inclusion and exclusion criteria and our rationale for these have been detailed in our published protocol.^27^ The trial was conducted in accordance with the Declaration of Helsinki^28^ and Good Clinical Practice guidelines.^29^ All participants provided written informed consent prior to participation.

### Randomization

**Figure 1.**
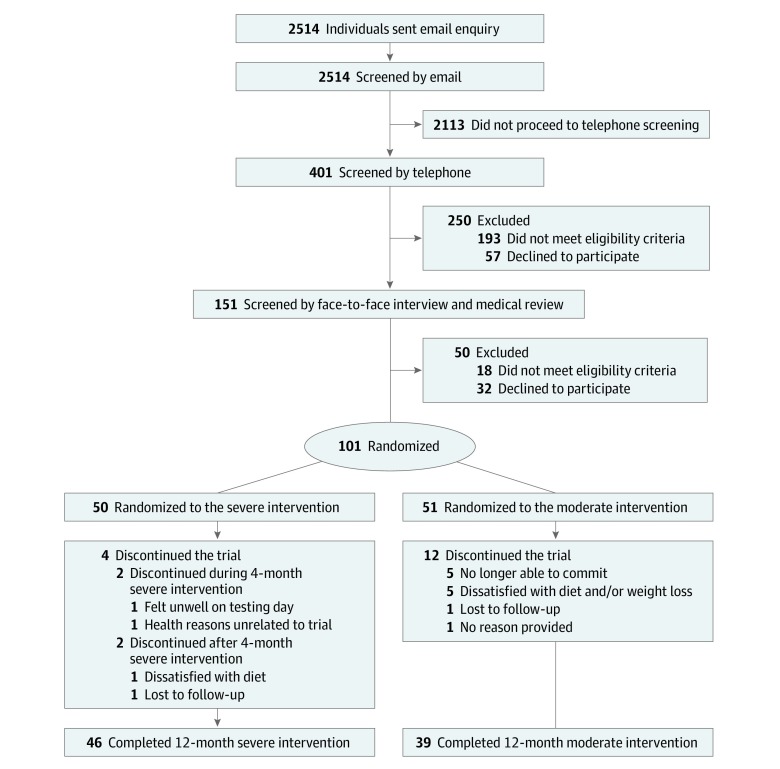
Trial Flow Diagram The moderate intervention involved a food-based diet with a 25% to 35% energy restriction for a total of 12 months (52 weeks). The severe intervention involved total diet replacement with 65% to 75% energy restriction for 4 months (16 weeks) or until a body mass index (calculated as weight in kilograms divided by height in meters squared) of no lower than 20 was reached, whichever came first, followed by moderate energy restriction until 12 months (52 weeks).

Participants were randomized (and enrolled) to either the severe or moderate intervention (Figure 1) using stratified permuted block randomization.^30^ Specifically, they were stratified by age (ie, 45 to <55 years and 55-65 years) and body mass index (ie, 30 to <35 and 35-40). Individuals in each of the 4 stratified groups were then randomized in blocks of 2 and with a 1:1 ratio into 1 of 2 interventions.^27,31^ To avoid bias, randomization was undertaken by 1 of us (A.S.) who had no contact with participants before randomization and was not involved in implementing the interventions. None of us who undertook screening or clinical testing (R.V.S., S.M., C.H., A.A.G, and H.A.F.) were aware of the method used for randomization, and we were not able to predict which intervention a particular participant would be randomized to.

### Procedures

The moderate intervention involved a moderate energy restriction of 25% to 35% relative to estimated energy expenditure for a total of 12 months (52 weeks). This was achieved using a food-based diet, with recommendations based on the Australian Guide to Healthy Eating.^32^ The guide provides recommendations on the average number of standard servings of the 5 core food groups (ie, vegetables, fruits, grains and cereals, meat and meat alternatives, and reduced fat dairy) that an individual should consume to meet nutritional requirements based on age and sex. To simplify adherence to the moderate intervention, we defined 6 food groups. The meat and meat alternative and reduced fat dairy food groups were collapsed into a proteins group, and starchy vegetables were incorporated into the grains and cereals group to form a carbohydrates group, while participants also had groups for vegetables, fruits, fats, and discretionary foods. The severe intervention involved a severe energy restriction of 65% to 75% relative to estimated energy expenditure for 4 months (16 weeks) or until a body mass index of no lower than 20 was reached, whichever came first. This was achieved using a total meal replacement diet (KicStart meal replacement shakes and soups from Prima Health Solutions) supplemented with a whey protein isolate (Beneprotein; Nestlé HealthCare Nutrition) to achieve the prescribed protein target (described later). This was followed by moderate energy restriction (ie, the moderate intervention) for the remaining period to 12 months (52 weeks). Both diets were individualized for each participant and were nutritionally sound. That is, the diet used in the moderate intervention was designed to meet nutrient requirements with minimum energy intake,^31^ while the severe intervention used a commercial total meal replacement product and supplemental protein that rendered it close to the recommended nutrient requirements.^33^ For both interventions, a protein intake of 1.0 g/kg of actual body weight per day was prescribed. Participants were encouraged to gradually increase step counts to a total of 8000 to 12 000 steps/d, including 30 to 60 min/d of moderate to vigorous physical activity.^34^ Although physical activity was encouraged, it was not supervised. Since the use of food diaries to measure adherence to the prescribed diet is difficult to assess because of missing dietary records and underreporting among participants with overweight and obesity,^35^ weight loss was used to monitor adherence to the diets.^36^ We expected approximately 1.5 to 2.5 kg/wk weight loss for participants in the severe intervention^6^ and approximately 0.5 to 1.0 kg/wk weight loss for participants in the moderate intervention.^37^ To increase adherence to the diet, participants attended individual dietary appointments with the trial dietitian approximately every 2 weeks for the first 26 weeks of the intervention (ie, at 1, 2, 4, 6, 8, 10, 12, 15, 16, 18, 20, 25, and 26 weeks relative to commencement of the dietary interventions, plus an extra appointment at 17 weeks for participants in the severe intervention during their transition to the moderate intervention) and then approximately every month until 52 weeks (ie, at 29, 33, 37, 41, 45, 51, and 52 weeks). The development process and rationale behind the dietary interventions for the TEMPO Diet Trial as well as full details of the dietary interventions have been published previously.^31^

### Outcomes

This article reports the primary outcome for the TEMPO Diet Trial (lean mass at 12 months after commencement of the intervention) as well as the other outcomes related to body composition, all of which are secondary outcomes. The body composition outcomes reported in this article are listed in Table 1, and the full list of outcomes for this trial are published in our trial protocol.^27^ All body composition outcomes were measured before the start of the intervention (0 months) and at 4, 6, and 12 months (with weight and waist and hip circumference additionally measured at 0.25 and 1 month) after commencing the interventions. Height was measured only at 0 months. All data were collected with participants lightly clothed (ie, in a close-fitting sports bra and leggings or, for MRI and MRS scans, in a gown and underpants only), without shoes, and with all metal jewelry, accessories, and electronic devices removed.

**Table 1.** Baseline Characteristics for All Participants, Completers and Noncompleters, in the TEMPO Diet Trial

Characteristic	Mean (SD)					
	Severe Group			Moderate Group		
	All Participants (n = 50)	Completers (n = 46)	Noncompleters (n = 4)	All Participants (n = 51)	Completers (n = 39)	Noncompleters (n = 12)
Age, y	58.0 (4.4)	58.2 (4.3)	55.4 (5.0)	58.0 (4.2)	57.7 (4.2)	58.9 (4.3)
Height, m	1.62 (0.06)	1.62 (0.06)	1.64 (0.04)	1.63 (0.05)	1.63 (0.06)	1.63 (0.04)
Weight, kg	90.1 (9.4)	89.5 (9.4)	96.8 (7.4)	92.4 (8.3)	92.2 (8.6)	93.0 (7.4)
Body mass index^a^	34.3 (2.5)	34.2 (2.4)	36.0 (2.5)	34.6 (2.5)	34.6 (2.7)	34.8 (2.1)
**Lean Tissues**						
Whole-body lean mass, kg^b^	44.3 (4.9)	44.1 (5.0)	46.5 (3.6)	44.8 (4.0)	44.5 (4.1)	45.7 (3.7)
Thigh muscle area, cm^2^^c^	111.4 (15.1)	110.1 (14.6)	126.2 (14.0)	109.7 (13.4)	108.9 (13.4)	112.8 (13.5)
Muscle strength, kg						
Dominant hand^d^	29.80 (6.31)	29.27 (6.10)	35.75 (7.27)	30.00 (4.70)	29.97 (4.45)	30.08 (5.66)
Nondominant hand	27.90 (6.00)	27.39 (5.69)	33.75 (7.27)	27.90 (4.42)	28.03 (4.59)	27.80 (3.97)
**Bone Mineral Density**						
Total hip, g/cm^2^^e^	0.988 (0.097)	0.980 (0.088)	1.079 (0.158)	0.972 (0.107)	0.973 (0.106)	0.967 (0.116)
Femoral neck, g/cm^2^^e^	0.810 (0.089)	0.803 (0.078)	0.882 (0.714)	0.815 (0.097)	0.821 (0.097)	0.793 (0.101)
Lumbar spine, g/cm^2^	1.001 (0.112)	0.994 (0.112)	1.080 (0.091)	1.019 (0.125)	1.004 (0.122)	1.066 (0.126)
Whole body, g/cm^2^^b^	1.093 (0.077)	0.089 (0.077)	1.139 (0.054)	1.100 (0.088)	1.089 (0.075)	1.133 (0.117)
**Fat Mass and Distribution**						
Waist circumference, cm	108.3 (7.3)	108.4 (7.3)	107.8 (8.2)	108.8 (7.0)	109.1 (7.5)	107.6 (4.8)
Hip circumference, cm	118.6 (7.0)	117.9 (6.7)	126.9 (6.1)	121.3 (6.6)	121.3 (6.2)	121.3 (8.0)
Ratio of waist to hip circumference^f^	0.915 (0.061)	0.921 (0.059)	0.850 (0.054)	0.898 (0.060)	0.901 (0.059)	0.891 (0.065)
Whole-body fat mass, kg^b^	42.2 (5.6)	41.8 (5.5)	46.7 (5.7)	43.5 (5.9)	43.5 (5.9)	43.4 (6.1)
Abdominal adipose tissue, cm^3^						
Subcutaneous adipose tissue^c^^,^^g^	12 006 (3028)	11 833 (2981)	14 544 (3080)	12 176 (2508)	12 005 (2620)	12 811 (2030)
Visceral adipose tissue^c^	4544 (1702)	4604 (1705)	3884 (1756)	5123 (1954)	5074 (2002)	5296 (1854)
Intrahepatic lipid, %^h^	16.7 (16.9)	15.4 (15.3)	29.6 (27.8)	22.4 (19.1)	23.9 (20.8)	17.1 (11.2)
Thigh fat area, cm^2^						
Subcutaneous fat area^c^	154.4 (28.9)	152.0 (26.4)	181.6 (45.0)	164.6 (43.4)	165.1 (36.0)	162.8 (65.6)
Subfascial fat area^c^	10.1 (4.1)	9.9 (4.0)	12.8 (4.2)	11.1 (4.3)	11.3 (4.6)	10.5 (2.9)
Intermuscular fat area^c^	6.07 (2.09)	6.00 (2.16)	6.92 (0.64)	6.78 (2.03)	6.82 (1.90)	6.63 (2.53)

Abbreviation: TEMPO, Type of Energy Manipulation for Promoting Optimum Metabolic Health and Body Composition in Obesity.

^a^ Calculated as weight in kilograms divided by height in meters squared.

^b^ Data for 2 participants in the moderate group who completed the study are missing because of machine failure.

^c^ Data for 2 participants in the severe group who completed the study and 1 in the moderate group who did not complete the study are missing because they did not undergo magnetic resonance imaging scan or magnetic resonance spectroscopy.

^d^ Data for 1 participant in the severe group who completed the study are missing because the participant underwent thumb tendon surgery, and data for 1 participant in the moderate group who completed the study are missing because the participant had scaphoid fracture.

^e^ Data for 1 participant in the moderate group who completed the study are missing because the scan could not be analyzed.

^f^ Calculated as waist circumference divided by hip circumference.

^g^ Data for 1 participant in the severe group who did not complete the study and 3 participants in the moderate group (2 completers, 1 noncompleter) are missing because the scan was outside the window of analysis.

^h^ Data for 7 participants in the severe group who completed the study and 11 participants in the moderate group (8 completers; 3 noncompleters) are missing because the scans were invalid and unable to be accurately analyzed.

Dual-energy x-ray absorptiometry (DXA), using a Discovery W bone densitometer (Hologic), was used to assess whole-body lean mass, whole-body fat mass, and BMD of the total left hip and anterior posterior lumbar spine (L1-L4).^38^ A hydraulic hand dynamometer (Jamar, Model 5030J1; Patterson Medical) was used to assess handgrip strength of the dominant and nondominant hands. For 2 participants who were ambidextrous, the right and left hand were designated the dominant and nondominant hand, respectively. Waist and hip circumferences were measured to the nearest 0.1 cm using a narrow, flexible, and inelastic steel tape (Lufkin W606PM; Apex Tool Group). Waist circumference was measured at the midaxillary line (ie, at the halfway point between the bony landmarks of the lowest rib and the top of the iliac crest), while the hip circumference measurement was taken at the point of greatest protuberance of the participant’s buttocks when viewed from the side. The ratio of waist to hip circumference was calculated by dividing waist circumference by hip circumference. A 3-T MRI scanner (Discovery MR750; GE Healthcare) was used to assess abdominal fat volume (subcutaneous and visceral adipose tissue from the diaphragm to the pelvis, with a slice thickness of 10 mm and an interslice gap of 10 mm) as well as thigh muscle area and thigh fat area (subcutaneous, subfascial, and intermuscular, with the median slice between the base of the femoral head and midpatella selected for analysis unless there were an even number of slices between these points, in which case the inferior of the 2 middle slices was selected). Intrahepatic lipid was measured by volume localized proton MRS (^1^H-MRS) and calculated as methylene peak area + methyl peak area × 100 / water peak area, corrected for T_2_ effects. Accelerometers (SenseWear Pro Armband; BodyMedia Inc) were used to assess physical activity.^39^ Physical activity data will not be presented in this article; however, it was used as a covariate in our analysis. Physical activity data were only included as a covariate in the analysis if participants wore the accelerometer for a minimum of 5 of 7 designated days for at least 85% of each 24-hour day. Mean physical activity across the days the accelerometer was worn was then reported as metabolic equivalents of task (equal to total energy expenditure divided by resting energy expenditure; calculated by SenseWear Professional Software version 7.0 [BodyMedia Inc]).

### Statistical Analysis

For our primary outcome of whole-body lean mass at 12 months after intervention commencement, we calculated that a target sample size of 100 participants would provide a power of 90% at a 2-sided α level of 5%, allowing for up to 20% attrition as seen in previously published weight loss interventions.^40,41,42^ Notably, attrition at 12 months in our trial was 16%, which fell within the attrition prediction used in our sample size calculation.

Statistical analyses were performed using SPSS statistical software version 24 for Windows (IBM Corp). Statistical significance was accepted as *P* < .05, and tests were 2-tailed. Fisher exact tests were used to compare categoric demographic characteristics (ie, race) and attrition between groups (ie, severe vs moderate). To compare continuous variables between groups at baseline (0 months), Mann-Whitney tests were used. All continuous variables were assessed for normality before analysis using histograms and *P*-*P* plots. Where data were not normally distributed (ie, intrahepatic lipid and subfascial thigh fat area), a natural log transformation was applied to obtain a normal distribution.

To compare longitudinal changes between groups, intention-to-treat analysis was performed using data from all participants originally randomized, using random-effects linear mixed models. Mixed-model analyses were used instead of standard repeated measures analysis of variance because of the likelihood that there would be dropouts and missed visits that precluded the use of the classic approach.^43^ Therefore, this model allowed for participants to have partial missing data and still be included without imputation. Intervention group and time (ie, 0, 4, 6, and 12 months for all parameters, except waist circumference, hip circumference, and waist-to-hip ratio, which were assessed at all of these points plus at 0.25 and 1 month) were included as fixed effects, and participant was included as a random effect. For all outcomes, baseline values of the relevant variable were added as a covariate in the analysis. As the degree of physical activity is known to influence measures of body composition, physical activity (expressed in metabolic equivalents of task) was added as a covariate, as measured at each point. When the overall *P* value for the interaction between group and time was less than .05, comparisons between groups at each point were made, using Bonferroni adjustments to correct for multiple comparisons. As a secondary analysis, the model was further adjusted for weight at each point, because lighter individuals are known to have lower whole-body lean mass and BMD than heavier individuals.^44,45^ This was to determine if there was an effect of the interventions beyond that which would be expected due to weight loss.

Within-group changes were analyzed with repeated measures linear-mixed models. Intervention group and time were included in the model as fixed effects and time as a repeated measure, with physical activity (metabolic equivalents of task) added as a covariate. Maximum-likelihood estimation was used, and an unstructured covariance matrix was specified. When the overall *P* value for the interaction between group and time was less than .05, comparisons between time points and baseline (0 months) within each group were made, using Bonferroni adjustments to correct for multiple comparisons.

## Results

Participants were recruited between March 2013 and July 2016, and 101 participants were randomized to the trial (Figure 1). Participants had a mean (SD) age of 58.0 (4.2) years, a mean (SD) weight of 90.8 (9.1) kg, and a mean (SD) body mass index of 34.4 (2.5). At baseline (0 months), there were no differences between groups in age, race (severe: 47 of 50 [94.0%] white participants; moderate: 48 of 51 [94.1%] white participants), or outcome variables (Table 1). Overall, 85 of 101 (84.2%) completed the 12-month intervention (46 of 50 [92.0%] in the severe group vs 39 of 51 [76.5%] in the moderate group). There were 3-fold fewer participants discontinuing the trial for the severe group compared with the moderate group (4 vs 12 participants; *P* = .05) (Figure 1).

### Weight

**Figure 2.**
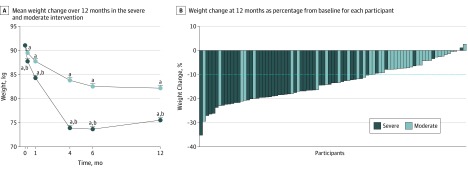
Weight Changes in Postmenopausal Women With Obesity During the Type of Energy Manipulation for Promoting Optimum Metabolic Health and Body Composition in Obesity (TEMPO) Diet Trial A, Weight data presented as estimated marginal means, ie, group means after controlling for covariates. B, Weight change at 12 months as percentage change from baseline for each participant in the severe and moderate groups. The dotted line indicates 10% weight loss. For both panels, the severe intervention included 50 participants and the moderate intervention, 51. ^a^*P* < .001 vs baseline value within group. ^b^*P* < .05 vs the moderate group at that point.

The severe group had a significantly lower weight than the moderate group at all points after baseline (effect size, −6.6 kg; 95% CI, −8.2 to −5.1 kg; estimated marginal mean at 12 months: −15.3 [95% CI, −18.1 to −12.5] vs −8.4 [95% CI, −11.4 to −5.4] kg; *P* < .001) (Figure 2A and Table 2). Both groups had significant decreases in weight at all points compared with baseline. At 12 months, 41 of 46 participants (89.1%) in the severe intervention who remained in the study had lost at least 10% of their baseline weight compared with 14 of 39 participants (35.9%) in the moderate intervention who remained in the study (*P* < .001) (Figure 2B). Assuming participants who dropped out of the study did not achieve at least 10% weight loss, then 41 of 50 participants (82.0%) in the severe group lost at least 10% of baseline weight compared with 14 of 51 participants (27.5%) in the moderate group (*P* < .001). This represents a 2.5-fold to 3.0-fold greater likelihood of losing a clinically significant amount of weight (ie, 10%) with the severe vs moderate intervention.

**Table 2.** Changes From Baseline for Body Composition With the Severe and Moderate Energy Restriction Interventions of the TEMPO Diet Trial

Measurement	No.	Severe Group, Estimated Marginal Mean (95% CI)	No.	Moderate Group, Estimated Marginal Mean (95% CI)	*P* Value^a^
Weight change, kg					
0.25 mo	48	−2.7 (−3.0 to −2.4)^b^	51	−1.5 (−1.8 to 1.2)^b^	.02
1 mo	48	−6.3 (−6.8 to −5.8)^b^	48	−3.1 (−3.6 to −2.6)^b^	<.001
4 mo	48	−17.4 (−18.9 to −16.0)^b^	43	−7.1 (−8.6 to −5.6)^b^	<.001
6 mo	48	−17.8 (−19.7 to −15.9)^b^	43	−8.5 (−10.5 to −6.6)^b^	<.001
12 mo	46	−15.3 (−18.1 to −12.5)^b^	39	−8.4 (−11.4 to −5.4)^b^	<.001
Weight change, % of baseline					
0.25 mo	48	−3.1 (−3.4 to −2.7)^b^	51	−1.6 (−1.9 to 1.2)^b^	.02
1 mo	48	−7.0 (−7.6 to −6.5)^b^	48	−3.4 (−4.0 to −2.8)^b^	<.001
4 mo	48	−19.6 (−21.3 to −17.9)^b^	43	−7.7 (−9.4 to −6.0)^b^	<.001
6 mo	48	−19.9 (−22.0 to −17.8)^b^	43	−9.3 (−11.4 to −7.1)^b^	<.001
12 mo	46	−17.3 (−20.3 to −14.3)^b^	39	−8.8 (−12.0 to −5.7)^b^	<.001
Body mass index change^c^					
0.25 mo	48	−1.05 (−1.17 to −0.93)^b^	51	−0.55 (−0.67 to −0.43)^b^	.02
1 mo	48	−2.39 (−2.57 to −2.21)^b^	48	−1.17 (−1.35 to −0.99)^b^	<.001
4 mo	48	−6.61 (−7.15 to −6.07)^b^	43	−2.67 (−3.22 to −2.13)^b^	<.001
6 mo	48	−6.73 (−7.44 to −6.02)^b^	43	−3.19 (−3.92 to −2.46)^b^	<.001
12 mo	46	−5.81 (−6.89 to −4.74)^b^	39	−3.17 (−4.31 to −2.02)^b^	<.001
Lean tissue change					
Whole-body lean mass, kg^d^					
4 mo	48	−3.7 (−4.4 to −2.9)^b^	42	−1.4 (−2.1 to −0.7)^b^	<.001
6 mo	47	−3.0 (−4.0 to −2.1)^b^	40	−1.9 (−2.9 to −1.0)^b^	.002
12 mo	45	−3.2 (−4.1 to −2.3)^b^	37	−2.1 (−3.1 to −1.2)^b^	.005
Thigh muscle area, cm^2^					
4 mo	46	−12.7 (−15.1 to −10.2)^b^	42	−3.3 (−5.5 to −1.0)^b^	<.001
6 mo	45	−8.7 (−11.1 to −6.4)^b^	40	−4.4 (−6.6 to −2.3)^b^	<.001
12 mo	43	−8.2 (−10.7 to −5.7)^b^	38	−3.9 (−6.5 to −1.4)^b^	<.001
Muscle strength change, kg					
Dominant hand					
4 mo	47	0.29 (−1.38 to 1.95)	43	−2.00 (−3.56 to −0.43)	NA^e^
6 mo	46	−0.36 (−2.02 to 1.33)	40	−1.05 (−2.63 to 0.53)	NA^e^
12 mo	42	−0.49 (−2.32 to 1.35)	39	−1.54 (−3.46 to 0.38)	NA^e^
Nondominant hand					
4 mo	48	−0.59 (−2.15 to 0.97)	43	−1.61 (−3.07 to −0.14)^f^	NA^e^
6 mo	47	−0.42 (−2.14 to 1.31)	41	−1.16 (−2.81 to 0.49)	NA^e^
12 mo	43	−1.06 (−2.91 to 0.79)	38	−1.83 (−3.78 to −0.13)	NA^e^
Bone mineral density change, g/cm^2^^d^					
Total hip					
4 mo	48	−0.018 (−0.029 to −0.006)^b^	42	−0.008 (−0.018 to 0.003)	.10
6 mo	48	−0.020 (−0.031 to −0.008)^b^	42	−0.008 (−0.019 to −0.002)	.06
12 mo	46	−0.032 (−0.045 to −0.029)^b^	38	−0.015 (−0.028 to −0.002)^f^	.002
Femoral neck					
4 mo	48	0.013 (−0.034 to 0.008)	42	−0.011 (−0.031 to 0.009)	.99
6 mo	48	−0.018 (−0.039 to 0.002)	42	−0.012 (−0.032 to 0.008)	.65
12 mo	46	−0.034 (−0.054 to −0.014)^b^	38	−0.020 (−0.040 to −0.001)	.23
Lumbar spine					
4 mo	47	−0.004 (−0.019 to 0.011)	41	−0.007 (−0.021 to 0.007)	.54
6 mo	48	−0.021 (−0.036 to −0.005)^g^	42	−0.011 (−0.025 to 0.003)	.33
12 mo	46	−0.033 (−0.046 to −0.014)^b^	38	−0.021 (−0.038 to −0.003)^f^	.27
Whole body					
4 mo	48	0.006 (−0.006 to 0.017)	42	−0.003 (−0.014 to 0.008)	.05
6 mo	47	0.004 (−0.007 to 0.015)	40	0.000 (−0.010 to 0.010)	.32
12 mo	45	−0.008 (−0.008 to 0.002)	37	−0.010 (−0.021 to 0.000)^f^	.38
Fat mass and distribution change					
Waist circumference, cm					
0.25 mo	48	−1.9 (−2.8 to −0.9)^b^	51	−0.3 (−1.2 to 0.7)	.04
1 mo	48	−4.6 (−5.7 to −3.5)^b^	48	−2.0 (−3.1 to −0.9)^b^	.004
4 mo	48	−15.2 (−17.1 to −13.2)^b^	43	−5.5 (−7.3 to −3.6)^b^	<.001
6 mo	48	−15.4 (−17.8 to −12.9)^b^	42	−6.1 (−8.6 to −3.6)^b^	<.001
12 mo	46	−14.3 (−17.3 to −11.3)^b^	38	−6.9 (−10.1 to −3.7)^b^	<.001
Hip circumference, cm					
0.25 mo	48	−1.2 (−2.2 to −0.1)^f^	51	−0.5 (−1.5 to 0.6)	.04
1 mo	48	−3.6 (−4.5 to −2.6)^b^	48	−1.9 (−2.8 to −0.9)^b^	.001
4 mo	48	−12.2 (−13.8 to −10.7)^b^	43	−5.6 (−7.0 to −4.1)^b^	<.001
6 mo	48	−13.2 (−15.2 to −11.2)^b^	42	−6.6 (−8.5 to −4.6)^b^	<.001
12 mo	46	−10.3 (−12.7 to −7.8)^b^	38	−6.2 (−8.9 to −3.5)^b^	<.001
Ratio of waist to hip circumference^h^					
0.25 mo	48	−0.002 (−0.040 to 0.036)	51	0.003 (−0.034 to 0.041)	.29
1 mo	48	−0.006 (−0.044 to 0.031)	48	0.004 (−0.034 to 0.041)	.30
4 mo	48	−0.027 (−0.065 to 0.012)	43	−0.003 (−0.040 to 0.004)	<.001
6 mo	48	−0.021 (−0.060 to 0.018)	42	0.004 (−0.035 to 0.043)	.001
12 mo	46	−0.038 (−0.075 to −0.001)^f^	38	−0.005 (−0.046 to 0.035)	<.001
Whole-body fat mass change, kg^d^					
4 mo	48	−11.4 (−12.6 to −10.1)^b^	42	−4.6 (−5.8 to −3.4)^b^	<.001
6 mo	47	−12.2 (−13.8 to −10.6)^b^	40	−5.5 (−7.1 to −3.9)^b^	<.001
12 mo	45	−10.2 (−12.1 to −8.4)^b^	37	−5.5 (−7.5 to −3.4)^b^	<.001
Abdominal adipose tissue change, cm^3^					
Subcutaneous					
4 mo	46	−3627 (−4188 to −3065)^b^	41	−1387 (−1915 to −859)^b^	<.001
6 mo	45	−4254 (−4964 to −3545)^b^	40	−1814 (−2498 to −1129)^b^	<.001
12 mo	43	−3391 (−4220 to −2562)^b^	37	−1624 (−2511 to −736)^b^	<.001
Visceral					
4 mo	46	−1948 (−2250 to −1646)^b^	42	−719 (−999 to −439)^b^	<.001
6 mo	45	−2386 (−2736 to −2035)^b^	40	−983 (−1312 to −654)^b^	<.001
12 mo	42	−2379 (−2839 to −1919)^b^	38	−1077 (−1561 to −594)^b^	<.001
Intrahepatic lipid change^i^					
4 mo	45	0.25 (0.18 to 0.35)^b^	40	0.53 (0.38 to 0.73)^b^	<.001
6 mo	45	0.28 (0.19 to 0.40)^b^	39	0.49 (0.34 to 0.70)^b^	<.001
12 mo	41	0.32 (0.22 to 0.46)^b^	35	0.40 (0.27 to 0.61)^b^	.04
Thigh fat area change, cm^2^					
Subcutaneous					
4 mo	46	−41.4 (−47.4 to −35.3)^b^	42	−19.2 (−24.9 to −13.6)^b^	<.001
6 mo	45	−49.7 (−57.2 to −42.2)^b^	40	−26.5 (−33.8 to −19.3)^b^	<.001
12 mo	43	−38.9 (−48.6 to −29.2)^b^	38	−24.0 (−34.2 to −13.8)^b^	<.001
Subfascial^i^					
4 mo	46	0.83 (0.77 to 0.89)^b^	42	0.95 (0.89 to 1.02)	<.001
6 mo	45	0.76 (0.71 to 0.83)^b^	40	0.92 (0.86 to 0.99)^b^	<.001
12 mo	43	0.79 (0.73 to 0.85)^b^	38	0.89 (0.82 to 0.96)^b^	<.001
Intermuscular					
4 mo	46	−1.70 (−2.10 to −1.30)^b^	41	−0.91 (−1.32 to −0.49)^b^	<.001
6 mo	45	−2.09 (−2.54 to −1.63)^b^	40	−1.19 (−1.66 to −0.71)^b^	<.001
12 mo	43	−1.80 (−2.31 to −1.29)^b^	38	−1.05 (−1.58 to −0.51)^b^	.001

Abbreviations: NA, not applicable; TEMPO, Type of Energy Manipulation for Promoting Optimum Metabolic Health and Body Composition in Obesity.

^a^ *P* values for comparison between the severe and moderate interventions at each point.

^b^ *P* < .001 vs baseline for that group. For within-group comparisons between follow-up and baseline values, a repeated-measures linear mixed model was used.

^c^ Calculated as weight in kilograms divided by height in meters squared.

^d^ Measured by dual-energy x-ray absorptiometry.

^e^ Group by time interaction was not significant; therefore, a pairwise comparison was not carried out.

^f^ *P* < .05 vs baseline for that group. For within-group comparisons between follow-up and baseline values, a repeated-measures linear mixed model was used.

^g^ *P* < .01 vs baseline for that group. For within-group comparisons between follow-up and baseline values, a repeated-measures linear mixed model was used.

^h^ Calculated as waist circumference divided by hip circumference.

^i^ Geometric mean ratio (95% CI).

### Lean Tissues

**Figure 3.**
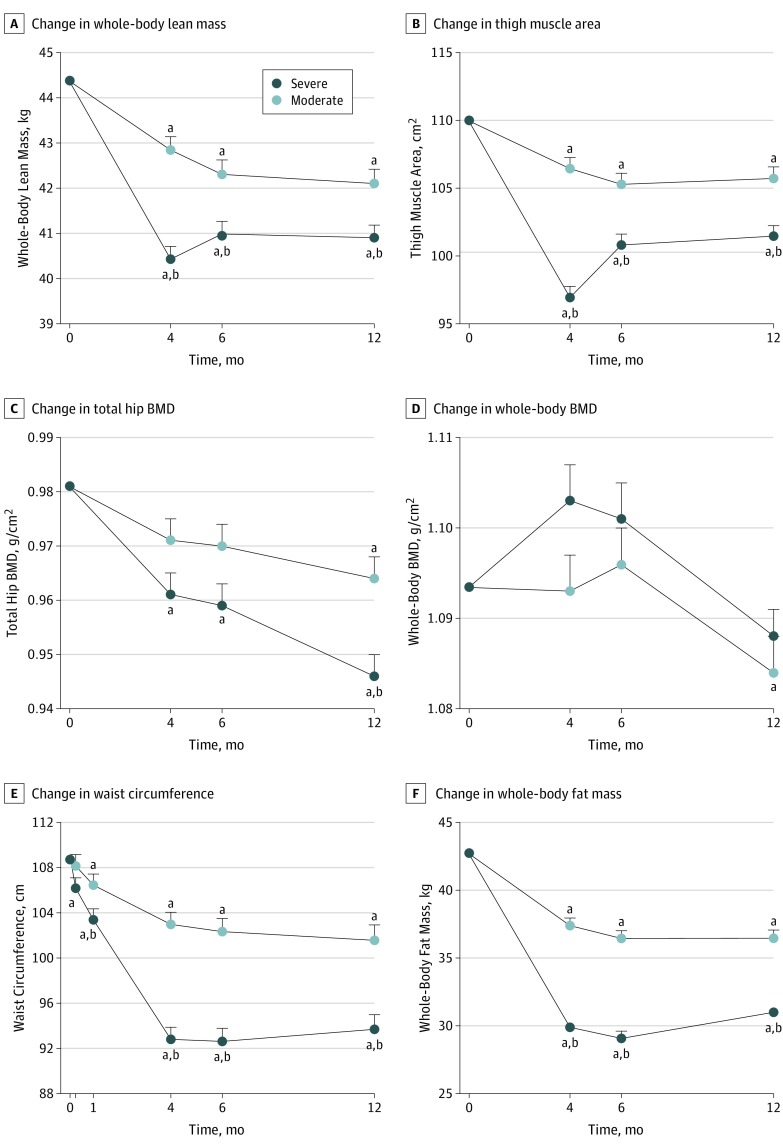
Effect of Severe vs Moderate Energy Restriction on Body Composition in Postmenopausal Women With Obesity Data are presented as estimated marginal means (ie, group means after controlling for covariates). Baseline values were the covariates in the statistical analysis model. Whiskers indicate SEs of the means. BMD indicates bone mineral density. ^a^*P* < .05 vs baseline value within group. ^b^*P* < .05 vs the moderate group at that point.

The severe group had significantly lower values of whole-body lean mass compared with the moderate group at all points after baseline (effect size, −1.2 kg; 95% CI, −2.0 to −0.4 kg; estimated marginal mean at 12 months: −3.2 [95% CI, −4.1 to −2.3] kg vs −2.1 [95% CI, −3.1 to −1.2] kg; *P* = .005) (Figure 3A and Table 2). Similar findings were seen when the analysis was run for only participants who completed the trial. After adjusting for body weight at each point, the severe group still had significantly lower values of whole-body lean mass than the moderate group at 4 months but not at 6 or 12 months. Both groups had significant decreases in whole-body lean mass at all points compared with baseline (estimated marginal mean at 12 months, severe group: −3.2 [95% CI, −4.1 to −2.3] kg; moderate group: −2.1 [95% CI, −3.1 to −1.2] kg; *P* = .005).

Similar to whole-body lean mass, the severe group had significantly lower thigh muscle area than the moderate group at all points after baseline (effect size, −4.2 cm^2^; 95% CI, −6.5 to −1.9 cm^2^; estimated marginal mean at 12 months: −8.2 [95% CI, −10.7 to −5.7] cm^2^ vs −3.9 [95% CI, −6.5 to −1.4] cm^2^; *P* < .001) (Figure 3B and Table 2). After adjusting for body weight at each point, the severe group still had significantly lower values of thigh muscle area compared with the moderate group at 4 months but not at 6 or 12 months. Both groups had significant decreases in thigh muscle area at all points compared with baseline.

There were no significant differences between the severe and moderate groups in dominant or nondominant handgrip strength (estimated marginal mean at 12 months for the dominant hand: −0.49 [95% CI, −2.32 to 1.35] kg vs −1.54 [95% CI, −3.46 to 0.38] kg; nondominant hand: −1.06 [95% CI, −2.91 to 0.79] kg vs −1.83 [95% CI, −3.78 to −0.13] kg) (Table 2; eFigure, A in Supplement 2). The severe group did not show any significant changes from baseline in either dominant or nondominant handgrip strength. The moderate group had a significant decrease from baseline to 4 months in the nondominant hand (estimated marginal mean, −1.61; 95% CI, −3.07 to −0.14; *P* = .02), but no significant changes from baseline in the dominant hand (Table 2; eFigure, A in Supplement 2).

### Bone Mineral Density

The severe group had significantly lower total hip BMD than the moderate group at 12 months (effect size, −0.017 g/cm^3^; 95% CI, −0.029 to −0.005 g/cm^2^; estimated marginal mean: −0.032 [95% CI, −0.045 to −0.029] g/cm^2^ vs −0.015 [95% CI, −0.028 to −0.002] g/cm^2^; *P* = .002), but there were no significant differences between groups in lumbar spine BMD (estimated marginal mean: −0.033 [95% CI, −0.046 to −0.014] g/cm^2^ vs −0.021 [95% CI, −0.038 to −0.003] g/cm^2^; *P* = .27) or whole-body BMD (estimated marginal mean: −0.008 [95% CI, −0.008 to 0.002] g/cm^2^ vs −0.010 [95% CI, −0.021 to 0.000] g/cm^2^; *P* = .38) (Figure 3C and D and Table 2; eFigure, B in Supplement 2). After adjusting our analyses for weight at each point, there was still a significantly lower total hip BMD in the severe group compared with the moderate group at 12 months. Both groups had significant decreases from baseline (Figure 3C and D and Table 2; eFigure, B in Supplement 2). Specifically, the severe group had significant decreases from baseline in total hip BMD at all points and in lumbar spine BMD at 6 and 12 months, while the moderate group had significant decreases from baseline in BMD in all 3 sites at 12 months.

There was a significant increase in the number of participants with osteopenia (defined as a *T*-score of –1 to –2.5)^46^ at the femoral neck in the severe group but not the moderate group (severe: 0 months, 8 of 50 [16.0%]; 12 months, 18 of 46 [39.1%]; *P* = .04; moderate: 0 months, 12 of 50 [24.0%]; 12 months, 11 of 38 [28.9%]; *P* *>* .99). In contrast, in the total hip there was no difference between the severe and moderate groups in the number of participants with osteopenia at 0 or 12 months (severe: 0 months, 0 of 50; 12 months, 1 of 46 [2.2%]; moderate: 0 months, 3 of 50 [6.0%]; 12 months, 4 of 38 [10.5%]; *P* > .99). There were no participants with osteoporosis (defined as a *T*-score of –2.5 or less)^46^ at 0 or 12 months in the severe or moderate groups.

### Fat Mass and Distribution

Differences between the 2 groups in waist and hip circumference, or the ratio thereof, were observed starting at 1 to 4 months. Indeed, the severe group had significantly lower waist and hip circumferences compared with the moderate group at all points after baseline (estimated marginal mean of waist circumference at 12 months: −14.3 [95% CI, −17.3 to −11.3] cm vs −6.9 [95% CI, −10.1 to −3.7] cm; *P* < .001; hip circumference at 12 months: −10.3 [95% CI, −12.7 to −7.8] cm vs −6.2 [95% CI, −8.9 to −3.5] cm; *P* < .001) (Figure 3E and Table 2), and the severe group had significantly lower values of waist to hip ratio than the moderate group at 4, 6, and 12 months, but not at 0.25 or 1 month (estimated marginal mean at 12 months: −0.038 [95% CI, −0.075 to −0.001] vs −0.005 [95% CI, −0.046 to 0.035]; *P* < .001) (Table 2). Both groups had significant decreases in waist and hip circumference at all points compared with baseline, except at 0.25 months for the moderate group. The severe group but not the moderate group had significant reductions in the ratio of waist to hip circumference at 12 months compared with baseline. Moreover, compared with the moderate group, the severe group had significantly lower whole-body fat mass (effect size, −5.5 kg; 95% CI, −7.1 to −3.9 kg; estimated marginal mean at 12 months: −10.2 [95% CI, −12.1 to −8.4] kg vs −5.5 [95% CI, −7.5 to −3.4] kg; *P* < .001) (Figure 3F and Table 2), abdominal subcutaneous and visceral adipose tissue volumes (subcutaneous adipose tissue: effect size, −1890 cm^3^; 95% CI, −2560 to −1219 cm^3^; estimated marginal mean at 12 months, −3391 [95% CI, −4220 to −2562] cm^3^ vs −1624 [95% CI, −2511 to −736] cm^3^; *P* < .001; visceral adipose tissue: effect size, −1389 cm^3^; 95% CI, −1748 to −1030 cm^3^; estimated marginal mean at 12 months, −2379 [95% CI, −2839 to −1919] cm^3^ vs −1077 [95% CI, −1561 to −594] cm^3^; *P* < .001), intrahepatic lipid (geometric mean ratio at 12 months: 0.32 [95% CI, 0.22 to 0.46] vs 0.40 [95% CI, 0.27 to 0.61]; *P* = .04), thigh subcutaneous adipose tissue area (estimated marginal mean at 12 months: −38.9 [95% CI, −48.6 to −29.2] cm^2^ vs −24.0 [95% CI, −34.2 to −13.8] cm^2^; *P* < .001) as well as thigh subfascial fat area (geometric mean ratio at 12 months: 0.79 [95% CI, 0.73 to 0.85] vs 0.89 [95% CI, 0.82 to 0.96]; *P* < .001) and intermuscular fat area (estimated marginal mean at 12 months: −1.80 [95% CI, −2.31 to −1.29] cm^2^ vs −1.05 [95% CI, −1.58 to −0.51] cm^2^; *P* < .001) at all points after baseline (Table 2; eFigure, C-H in Supplement 2). Both groups had significant decreases from baseline in all these parameters.

### Adverse Events

There were 8 adverse events (6 in the severe group, all related or possibly related to the intervention, and 2 in the moderate group, neither related to the intervention). These adverse events were reported by participants to the research team or occurred in the clinic during the clinical testing day. No adverse event was considered serious. In the severe intervention, the adverse events were hemorrhoids (2 participants [4.0%]), gallstones (2 participants [4.0%]), and hair loss (2 participants [4.0%]). In the moderate intervention, there were 2 episodes of migraine (1 participant [2.0%]), probably precipitated by fasting prior to clinical testing. This participant had a history of migraines and continued with the trial but only underwent outcome measurements that did not require fasting.

## Discussion

This randomized clinical trial demonstrated that, compared with moderate energy restriction over a 12-month period, severe energy restriction resulted in the following: (1) approximately 1.5 times as much loss of whole-body lean mass and thigh muscle area, although these losses were proportional to the amount of weight lost; (2) no difference in handgrip strength; (3) approximately twice as much weight loss (with participants 2.5-3 times more likely to lose 10% of their initial weight); (4) approximately twice as much total fat loss; and (5) a healthier fat distribution, as indicated by approximately twice the loss of waist circumference, waist-to-hip circumference ratio, and abdominal adipose tissue volume (subcutaneous and visceral). Participants in the severely energy-restricted intervention were also 3 times less likely to discontinue the trial compared with those in the moderately energy-restricted intervention. This is possibly because the large and rapid weight loss associated with severe energy restriction has been shown to be encouraging and because the total meal replacement diet used to achieve it is simple and convenient.^47^ These striking findings were offset by an approximately 2.5-fold greater loss of total hip BMD with severe energy restriction compared with moderate energy restriction, a difference not accounted for by the greater weight loss.

In this trial, the participants in the moderate group experienced approximately 1.3% reduction in total hip BMD after 12 months, similar to the annual rate of BMD loss at the hip in the early postmenopausal years (approximately 1.0%-1.4%).^48^ However, after the 12-month severe intervention, total hip BMD loss (approximately 3.3%) was 2.4 to 3.3 times higher than these annual BMD losses. Interestingly, the decrease in BMD continued over the whole 12 months of the severe intervention, even though weight loss had plateaued by 6 months. This occurred despite a dietary protein prescription of 1 g/kg of actual body weight per day in both groups^31,49^ and despite the fact that the total meal replacement products used in this trial contained more than the Australian recommended dietary intake for vitamin D and calcium for women aged 51 to 70 years.^33^ This loss of BMD may have been exacerbated because of the population selected for this trial (women with obesity who were ≥5 years postmenopausal). For example, in a study where men and women with obesity were prescribed a total meal replacement diet until they reached 15% weight reduction in 3 to 6 months, all participant groups exhibited significant decreases in body weight at 2 years, and both groups showed loss of BMD over 2 years, but this loss was statistically significant only among women.^50^ This suggests that women may have a greater propensity to lose BMD following weight loss. Research also suggests that BMD loss may be exacerbated in postmenopausal women (as in this trial) compared with women who are still in the perimenopausal transition.^48^

The consequences of accelerated BMD loss with a severely energy-restricted dietary obesity treatment are clinically concerning, especially if BMD loss continues beyond the 12-month intervention, because it has been linked to an increased risk of osteoporosis and fragility fracture.^51,52^ However, this bone loss must be considered in light of the beneficial effects of substantial weight loss on other health outcomes and health care costs. For example, although a 3% to 5% loss of initial weight has generally been accepted as being clinically significant,^11,53^ recent research shows that greater weight losses, ie, of 7.7%, 10%, 15%, or 20%, dose-dependently improve health outcomes.^54,55,56,57^ In addition, if treated effectively, the costs of obesity-related health complications would be significantly reduced.^2^ Thus, implementing effective obesity treatments is essential to reducing obesity-related comorbidities and the associated costs. Thus, while the current trial should not discourage the use of total meal replacement diets as a treatment for obesity in postmenopausal women, further investigation is needed to determine the long-term consequences of the associated BMD loss on health outcomes such as osteoporotic fractures and to determine how BMD losses could be prevented in this population during and after these diets.

### Strengths and Limitations

Strengths of this study include the 12-month randomized clinical trial design and the criterion-standard techniques used for the assessment of body composition, notably our primary outcome of lean mass. Another strength is that data variability was reduced by analysis of all data by a single researcher (ie, S.M. for DXA, A.L.W.-T. for MRI, and S.E.K. for MRS). There are, however, some limitations that must be noted, 1 of which is the technical limitation of DXA, which can only measure 2 tissue types at any time (eg, bone and soft tissue). Thus, being a 2-compartment model for the determination of body composition, a possible confounder is that the DXA analysis assumes a constant hydration of lean soft tissue, which is not always true, as hydration varies with age, sex, and disease. To help control for this, all participants were measured after an overnight fast (≥8 hours) and after voiding their bladder once at home before attending our clinical research facility and again when they arrived at the clinic. Another technical limitation of DXA is that changes in BMD observed with large weight losses might be exaggerated because of the varying amounts of soft tissue over time, which can result in unpredictable errors in DXA bone measurements, to up to 20%.^58^ In addition, DXA assesses bone quantity and not bone quality (eg, bone microarchitecture) or osteoporotic fracture incidence, and it is possible that, despite BMD loss, bone quality and strength may have been preserved in our participants. Despite these technical limitations, DXA remains the criterion standard and the only available test for measuring BMD in clinical practice as well as being an important predictor of osteoporotic fracture.^59,60^ A further limitation of our trial is that participants were predominantly white, which limits the generalizability of the findings to populations of other races.

## Conclusions

In this randomized clinical trial, severe energy restriction with a total meal replacement diet in postmenopausal women with obesity induced greater weight loss and approximately 1.5-fold as much loss of whole-body lean mass and thigh muscle area compared with moderate energy restriction over 12 months. While these losses of lean tissues were proportional to the amount of weight lost and while muscle strength (ie, handgrip strength) was unaffected by severe vs moderate energy restriction, there was an approximately 2.5-fold greater loss of total hip BMD with severe compared with moderate energy restriction, a difference not accounted for by the greater weight loss. Therefore, caution is necessary when implementing severe energy restriction in postmenopausal women with obesity, especially in those with osteopenia or osteoporosis, for whom concurrent bone-strengthening treatments (eg, muscle strengthening exercises) are recommended.

## Notes

Supplement 1. **Trial Protocol**

Supplement 2. **eFigure.** Effect of Severe vs Moderate Energy Restriction on Body Composition in Postmenopausal Women With Obesity

Supplement 3. **Data Sharing Statement**
