# Supplementary material for: Effect of Weight Loss via Severe vs Moderate Energy Restriction on Lean Mass and Body Composition Among Postmenopausal Women With Obesity: The TEMPO Diet Randomized Clinical Trial
Source: JAMA Netw Open. 2019 Oct 30;2(10):e1913733. doi: 10.1001/jamanetworkopen.2019.13733 (PMC6824325; doi:10.1001/jamanetworkopen.2019.13733)
Supplement: Supplement 1. — Trial Protocol [file jamanetwopen-2-e1913733-s001.pdf]

Effect of Weight Loss via Severe vs Moderate Energy Restriction on Lean Mass and Body Composition in Postmenopausal Women with Obesity: The TEMPO Diet Randomized Clinical Trial

Radhika V Seimon, Anthony L Wild-Taylor, Shelley E. Keating, Sally McClintock, Claudia Harper, Alice A Gibson, Nathan A Johnson, Hamish A Fernando, Tania P Markovic, Jackie Center, Janet Franklin, Peter Y Liu, Stuart M Grieve, Jim Lagopoulos, Ian D Caterson, Nuala M Byrne, Amanda Sainsbury

Contents

1 Protocol**....... 2**

1.1 Original protocol 2

1.2 Final protocol (link to publication)....... 15

1.3 Summary of changes from original protocol 16

1.3.1 Changes made before recruitment of first participant 16

1.3.2 Changes made after recruitment of first participant 18

2 Statistical analysis plan**....... *19***

2.1 Original statistical analysis plan 19

2.2 Final statistical analysis plan....... 19

2.3 Summary of changes from original statistical analysis plan 19

1 Protocol

1.1 Original protocol

HUMAN RESEARCH ETHICS COMMITTEE (HREC)

Effects of fast versus slow weight loss on fat, muscle & bone in postmenopausal women

PROTOCOL

OVERALL HYPOTHESES AND AIMS

The worldwide prevalence of obesity is increasing at an alarming rate and is a major risk factor for type 2 diabetes and other diseases. An important aspect of curbing the impact of the obesity epidemic is providing effective long-term treatments. In clinical practice, meal replacement programs such as very low energy diets (VLEDs) are used with increasing frequency. Although such programs are safe and effective in the short-term (up to one year), few studies have assessed their long-term safety and efficacy. While there is evidence that the ketosis associated with VLEDs attenuates the appetite stimulating effect of weight loss, the fact that weight loss, notably when induced by VLEDs, induces adaptive responses that would tend to inhibit loss of – and promote regain of – adiposity (particularly central adiposity) while decreasing lean body mass and muscle strength and compromising skeletal health is of concern. Such changes include decreases in metabolic rate and physical activity, and neuroendocrine alterations such as increased circulating concentrations of cortisol, decreased thyroid function and decreased circulating levels of insulin like growth factor-1. Because of the known deleterious effects of these changes on body composition, it is vital to assess whether VLEDs have negative effects that could ultimately increase the risk of conditions such as diabetes, cardiovascular disease, sarcopenia and osteoporosis. This question is of particular importance given that individuals may use VLEDs for several months at a time repeatedly over many years in order to manage their weight.

The overall aim of this project is to compare the consequences of weight loss via VLED versus less severe energy restriction on the strength of adaptive responses to energy restriction, as well as effects on adiposity (particularly central adiposity and hepatic fat), lean body mass, muscle size, muscle strength and bone mass for up to 3 years.

To this end, healthy obese postmenopausal women will be randomized to one of two weight loss interventions; one consisting of at least 12 months of lifestyle management with moderate energy restriction (~500 kcal deficit per day, ‘conventional diet’), and the other consisting of 3-5 months on a VLED followed by conventional diet or partial meal replacement for a total of at least 12 months.

SPECIFIC HYPOTHESES AND AIMS

Part 1: Effect of VLED versus conventional diet on parameters that affect body composition

Hypothesis: Whilst the ketogenic state induced by VLED abates some of the adaptive responses that can oppose weight loss, promote regain and impair body composition, VLED will nonetheless induce stronger overall adaptive responses to weight loss than conventional diet.

Aim: To compare the effects of VLED versus conventional diet in obese adults on appetite, physical activity, metabolic rate, neuroendocrine status, circulating gut hormone concentrations, markers of bone turnover and sympathetic reactivity up to 12 months after commencement of each intervention. Groups will be matched by the amount of weight or fat lost (at differing time points) as well as by duration of intervention (at different levels of weight or fat lost). We will also compare the magnitude of these parameters before and 1 week after transition from the ketogenic VLED to the non-ketogenic conventional diet, to determine the effect of ketosis on these responses.

Part 2: Effect of VLED versus conventional diet on body composition

Hypothesis: While VLED will promote greater initial weight and fat losses than conventional diet, it may also promote greater losses of lean body mass, muscle diameter, muscle strength and bone density that may not be recovered, and a greater propensity for fat regain, particularly abdominally.

Aim: To compare the effects of VLED versus conventional diet in obese adults on body weight, waist and hip circumference, adiposity (including central adiposity and hepatic fat), lean body mass, muscle diameter, muscle strength and bone density for 36 months after the start of interventions.

RESEARCH PLAN AND METHODOLOGY

Participants. This study will be performed in women because the estimated lifetime risk of osteoporotic fractures is 3-fold higher in women than in men (40% versus 13%)[49]. Additionally, while the prevalence of obesity is similar in both sexes, over 70% of the people presenting for treatment at the Metabolism & Obesity Services (Royal Prince Alfred Hospital) are women. One hundred (100) 45-65 year old women will be recruited, all of them sedentary (< 60 minutes physical activity / week) with class I or II obesity (30-40 kg.m^2). All women will be at least 5 years post-menopausal, to circumvent known effects of female sex hormone cycles and the menopausal transition on parameters under investigation. Eligibility for the study requires subjects to be euthyroid, non-diabetic, non-osteoporotic, non-smoking, ambulatory, weight stable for at least 6 months (± 2 kg) and not taking medications known to affect heart rate, body composition or bone mass (e.g. glucocorticoids or beta blockers), no anti-resorptive therapy within the last 3 years, and no alcohol or drug dependency. Participants must not have metal in the body (e.g. from a pacemaker), as this is contraindicated for magnetic resonance imaging and

magnetic resonance spectroscopy. Respondents will be interviewed by telephone and if classified as eligible based on height, weight and medical history, will be invited to attend an information session.

Participants will be randomised to VLED or conventional diet. Both interventions will be administered at the Metabolism & Obesity Services and overseen by Professor Ian Caterson and Dr Tania Markovic and Dr Janet Franklin. By having the interventions administered by actual clinical services rather than by our research laboratory, our findings will reflect effects of each diet as administered in the real world. We already know that both VLED and conventional diet result in average weight losses of approximately 20% and 12% of initial body weight after 12 months, respectively, and this study will demonstrate the effects of such real world clinical interventions on important parameters of long-term health and wellbeing, notably body composition and the factors that influence it.

The VLED consists of a 2,100 kJ (~500 kcal) per day program (Optifast®, Nestlé Nutritionals, Vevey, Switzerland), a high quality VLED with appropriate micronutrients that has been used extensively in the Metabolism & Obesity Services. Participants in the VLED arm will follow the VLED continuously for at least 3 months and for up to 5 months, or until a BMI of < 25 $kg.m^2$ is reached. As starting BMI is 30-40 $kg.m^2$, some volunteers on VLED may reach their target weight within 5 months. All volunteers on the VLED will then switch to either partial meal replacement for further weight loss if required (~1,500 kcal per day), or lifestyle intervention for weight maintenance. Meal replacements such as Optifast® are routinely used in this way, and many patients favour such ongoing usage. Two to three months on VLED is known to induce endocrine changes that have been shown in turn to adversely affect fat distribution, muscle function or bone density within 3 months as outlined above, and so using VLED for up to 5 months will be sufficient to reveal any effects on body composition, if present. The conventional diet is a lifestyle management program consisting of moderate kilojoule restriction (~500 kcal deficit per day) that has also been used extensively at the Metabolism & Obesity Services and published by Professor Ian Caterson[51]. Participants in both interventions will be advised to use pedometers and, after determination of baseline daily step counts, will be encouraged to gradually increase their physical activity in increments of 15 minutes per day of moderate physical activity such as walking (~1,500 steps per day).

Sample size. Our sample size of 100 women (50 on each diet) was selected based on the following power calculations and allowing for up to 20% attrition as we have seen in our previously published weight loss interventions[50,52-55]. Most stringently, even if there is only a 5% difference between groups in – for example – fat free mass at any time point, we have the statistical power to detect it. Indeed, to detect a 5% difference in fat free mass (e.g. 50.3 versus 53.0 kg, and assuming a variance of 5 kg), the effect size is 0.52, so at 90% power and P<0.05, it is necessary to study 39 women per group (50 – 50 x 20% attrition = 40). For other parameters under study, the samples sizes required to detect clinically meaningful differences between diets are smaller. For instance, based on studies in obese subjects that elicited a comparable weight loss to the 12-20% over 12 months that is expected in the current work, the effect size to detect differences between diets in the change in circulating thyroid hormone concentrations, bone mineral density and thigh muscle diameter are large (>0.8), indicating that a sample of 20 per group would provide 90% power to detect significant (P<0.05) differences between diets. From

previous studies we are not able to estimate potential differences between interventions for effects on appetite, but the effect size of energy deficit (and thus weight loss) on increasing appetite does appear to be dose-responsive (R=0.46, P<0.001)[56]. We can thus expect to detect a difference of moderate effect size for appetite with 30 participants per group. Based on comparable studies eliciting 12-20% weight loss in obese individuals, the effect size for differences between VLED & conventional diet with respect to changes in body weight, fat mass, waist circumference, visceral adiposity & resting metabolic rate in the first 3 months are very large (>1.1), indicating that a sample of only 5 per group will provide 90% power to detect differences between the diets at P<0.05. At later time points, differences between diets with respect to body weight and parameters of body composition such as fat free mass will be smaller, but as described at the start of this section our sample of 50 participants on each diet provides sufficient power to detect this. We have access to gold-standard techniques to measure each of the outcome variables in this project[50,52-55], thereby minimizing variability and further contributing to our power to detect clinically meaningful differences between diets.

Part 1: Effect of VLED versus conventional diet on parameters that affect body composition

Rationale. With the increasing use of VLEDs, it is important to compare the effects of ketogenic VLED and conventional diet directly on the adaptive responses to weight loss, since these parameters can significantly influence body composition and metabolic health. A suite of parameters will be investigated to identify possible mechanisms by which VLED and conventional diet may induce differential effects on body composition and muscle strength, thereby highlighting possible pathways for amelioration of any such differences. Additionally, these physiological responses will provide insights into reasons for lack of weight loss in some individuals.

Weight stabilization. At -0.5 months prior to commencement of the VLED or conventional diet as shown in the figure on the following page, volunteers will attend our clinical research facility for measurement of height (stretch stature), weight, waist and hip circumference and will then be instructed to maintain a steady weight (± 1 kg) until the first time point (time = 0). This step reduces variability in baseline measures because dynamic fluctuations in energy balance significantly affect many of the outcome measures in this study. To achieve weight stability, volunteers will be given loan of a set of precise bathroom scales and instructions on how to maintain their weight with the aid of daily weighing.

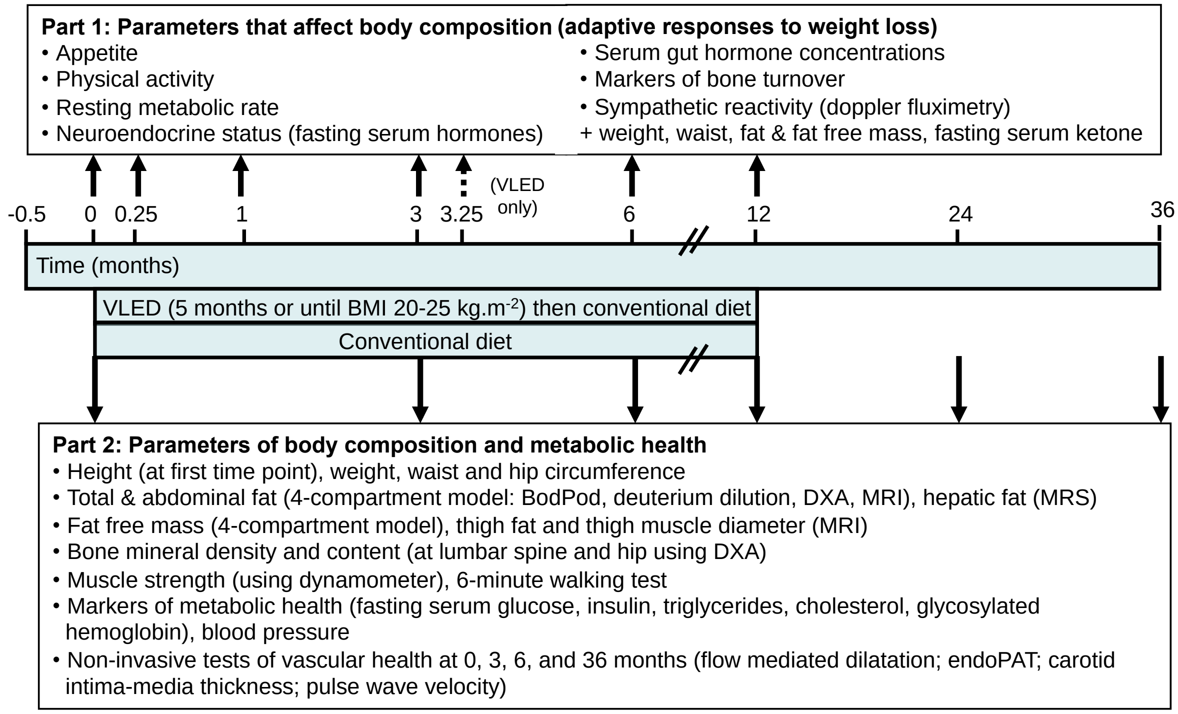

Time points and matching between groups. At 0, 0.25, 1, 3, 6 and 12 months after commencement of each diet, the adaptive responses to weight loss summarized in the above figure (top box) will be assessed. The necessity of measuring these parameters at 6 different time points is twofold. Firstly, as the magnitude of adaptive responses to weight loss increases with the amount of weight lost[11,12,15], multiple time points will enable us to compare subjects on the VLED and conventional diet after a similar degree of weight or fat loss. For the purposes of matching and standardization of results, at each of these time points we will also measure weight, waist circumference, as well as fat mass and fat free mass using the 4-compartment model (without DXA) as described below and as previously published by Professor Nuala Byrne[52,57,58]. Past experience from thousands of patients shows that weight losses after 0.25, 1 and 3 months on the VLED are similar to those induced by 1, 3 and 6-12 months on the conventional diet, respectively. In addition to matching for the degree of weight or fat loss, groups will also be compared at the same time points. A second reason for measuring these parameters at 6 time points is to determine the time of onset of various adaptive responses to weight loss, which will then be used to improve obesity interventions. For instance, we know that 2-3 months on a VLED induces strong adaptive responses that can promote weight regain and adverse body compositional changes. However, it is not known whether these responses are present for the entire duration of the VLED. If, for example, we discover that VLED (or conventional diet) induces changes such as increases in appetite and circulating cortisol levels or decreases in metabolic rate or serum IGF-1 that are seen only at the 3-month but not at the 1-month time point, then a future strategy might be to apply the intervention in intermittent bursts of less than 1 month, thereby attenuating the adaptive responses and their potentially deleterious effects on body composition.

Effect of ketosis on parameters that affect body composition. Participants in the VLED group will participate in this additional study. Following measurement of the adaptive responses to

weight loss at 3 months (at which point all participants will still be on the VLED, which results in ketosis due to its low carbohydrate and kilojoule content), participants will switch to a maintenance diet for 1 week followed by re-measurement at t = 3.25 months. Fasting serum concentrations of ketones such as β-hydroxybutyrate will also be determined before and after this transition.

Appetite. Participants will be provided with a fixed (200 kcal) liquid meal (Optifast®, Nestlé Nutritionals, Vevey, Switzerland) at 9:00 am. Subjective measures of motivation to eat will be measured immediately before, after and at 1, 2 and 3 hours thereafter using the Electronic Appetite Rating System as described by Professor Neil King[59].

Physical activity. Intensity and duration of daily physical activity will be measured using the Actigraph triaxial accelerometer worn on the waistband or on a belt. Accelerometry data will be collected during waking hours in the 7 days preceding each time point as previously described by Professor Nuala Byrne. Descriptive data will also be recorded in an activity diary, which will enable understanding of reasons for taking off the accelerometer (e.g. to swim or to sleep). Although all subjects will have the same standardized prescription to encourage physical activity, accelerometry and activity diary data combined with information from questionnaires (below) will provide insight as to possible effects of VLED or conventional diet on time spent exercising, in non-exercise activities or inactivity, and subjective measures of sleepiness.

Resting metabolic rate will be measured in the fasted state via indirect calorimetry using the TrueOne 2400 (Parvo Medics, Sandy, UT, USA) ventilated hood. Respired gases will be measured continuously for 30-min. VO_2 and VCO_2 will be converted to resting metabolic rate using the modified Weir equation as previously published by Professor Nuala Byrne[60-62].

Neuroendocrine status, circulating gut hormone and leptin concentrations will be assessed by determining concentrations of the following analytes in serum or plasma from overnight-fasted subjects: free T3, free T4, reverse T3, TSH, ACTH, cortisol, luteinizing hormone, follicle stimulating hormone, progesterone, oestradiol, IGF-1, PYY, ghrelin and leptin. Analyses will be done at Royal Prince Alfred Hospital or in-house using commercial radioimmunoassay kits. Associate Professor Amanda Sainsbury-Salis has extensive experience in collection and interpretation of endocrine data with respect to energy homeostasis[63,64]. As the hypothalamo-pituitary-thyroid, -adrenal, –gonadotropic and –somatotropic axes (as well as gut hormone and leptin levels) are influenced by weight reduction and are known to powerfully influence body composition if sustained for several weeks, these serial measurements will demonstrate whether there is any differential effect of VLED versus conventional diet on the concentrations of hormones that could adversely affect body composition, as well as the duration of any such changes. A suite of hormones will be measured, as it will allow a more accurate assessment of whether the activity of each axis has been differentially altered by either diet.

Bone. The following parameters will be assessed to determine possible mechanisms by which VLED or conventional diet may induce any effects on bone mass, as previously published by Associate Professor Jackie Centre[65]: serum calcium, phosphate, albumin, alkaline phosphatase, vitamin D, parathyroid hormone, osteocalcin, procollagen type 1 N-propeptide, C-telopeptide of type-I collagen, urinary hydroxyproline and 24-hour urinary calcium. All of these will be

performed at Royal Prince Alfred Hospital or in-house.

Sympathetic reactivity. Deep inspiration induces vasoconstriction in skin capillaries via sympathetic activation, and dynamic weight loss has been shown to inhibit this response[46], possibly by the same hypothalamic alterations that induce the various adaptive responses to weight loss. Sympathetic reactivity will thus be assessed by inspiration-induced vasoconstriction, as determined by laser Doppler fluximetry (MoorVMS-LDF® Laser Doppler Blood Flow Monitor, Moor, England) as previously published[46]. Because reduced sympathetic reactivity determined by this method predicts poor weight loss after bariatric surgery[46], and because this parameter is simple to measure and non-invasive, it may serve as a predictor of poor outcomes for weight loss or body composition in response to VLED or conventional diet. Such a measure could then be used in clinical practice to help identify individuals in likely need of more intensive support.

Questionnaires. Participants will complete validated instruments to assess quality of life (SF-36), depression symptoms (CES Depression Scale), Health Locus of Control (Multi-dimensional Health Locus of Control Questionnaire), sleep quality (Epworth Sleepiness Scale) & physical activity[66,67].

Database maintenance and data analysis. We have already developed a model database for use in our clinical research facility, and in collaboration with the NHMRC Clinical Trials Unit we are developing a database that can be used nationally. Database maintenance and data entry will be performed by Dr Radhika Seimon and will be directly overseen by Associate Professor Amanda Sainsbury-Salis. All data analyses will be performed using the SPSS statistical software package version 17.0 (SPSS Inc, Chicago, IL, USA). Repeated measures ANCOVA will be used for comparisons between all subjects on the VLED and the conventional diet, adjusting for confounding variables such as the amount of weight or fat lost or the length of time on the diet. Paired T-tests will be used to determine whether switching from the ketogenic VLED to the non-ketogenic maintenance diet alters the magnitude of any of the adaptive responses to weight loss.

Outcomes Part 1: This study will show for the first time whether VLED induces stronger adaptive responses to weight loss than conventional diet independent of the amount of weight or fat lost, and – by analysis with results in Part 2 – whether the magnitude of any of the adaptive responses predict outcomes for body composition and muscle diameter or strength. If so, then those adaptations could become targets for interventions to enhance weight loss and body composition in response to VLED or conventional diet. This work will also demonstrate whether ketones attenuate any of the adaptive responses to weight loss during VLED. If so, and if the magnitude of any adaptive responses predict outcomes for weight loss and body composition, then clinical interventions to ensure that patients on VLEDs maintain high circulating concentrations of ketones (e.g. by emphasizing the importance of compliance with the diet) could improve the effectiveness of VLEDs for the long-term management of overweight and obesity. An additional benefit of measuring multiple aspects of the adaptive response to weight loss at various time points is that it will enable subsequent improvements to both dietary interventions (e.g. by intermittent application of energy restriction), as well as early clinical identification of responders versus non-responders on both diets, thereby aiding the clinical management of obesity by targeting individuals with the greatest support needs.

Part 2: Effects of VLED versus conventional diet on body composition

Rationale. While there is strong evidence that VLEDs lead to adaptations that can have deleterious effects on body composition, and limited evidence that such adaptations may be stronger during VLED than conventional diets, no long-term randomised controlled trials have directly compared the effects of VLED or conventional diet on body composition (adiposity, including central adiposity and hepatic fat content, lean body mass, muscle diameter, muscle strength and bone density). This question is of utmost clinical significance given the importance of maintaining optimum body composition for reducing the risk of diabetes, cardiovascular disease, frailty and osteoporosis, and given the increasing use of VLEDs for the clinical management of overweight and obesity.

Parameters of body composition and metabolic health. At 0, 3, 6, 12, 24 and 36 months after commencement of VLED or conventional diet, parameters of body composition and metabolic health will be measured as outlined in the figure above (lower box). Our established gold-standard techniques for in-depth analysis of body composition, combined with the clinical facilities and equipment available to us as well as our expertise in the analysis of such data as described below will enable us to obtain definitive answers as to the effects of VLED versus conventional diet on body composition. In addition, we will also perform non-invasive tests of vascular health at 0, 3, 6, and 36 months after commencement of either diet, using methodology established by Dr Michael Skilton, in order to assess any differences between the two diets with respect to changes in cardiovascular risk. It is necessary to measure these parameters at multiple time points; whereas the diets will likely have differential beneficial or adverse effects on body composition after 3, 6 or 12 months, a question of vital relevance to long-term structural and metabolic health is whether any such differences are sustained for up to 24 and 36 months after commencement of either diet.

Measurements are weight, waist and hip circumference, total fat mass and fat free mass calculated with the gold standard 4-compartment model using independent measures of total body density (measured by plethysmography using a BodPod®, Life Measurement Inc, Concord, CA, USA), total body water (determined by deuterium dilution), and bone mineral content (determined by dual energy X-ray absorptiometry – DXA – using a Lunar Prodigy, Madison, WI, USA) as published by Professor Nuala Byrne[52,57,58]. In addition, abdominal adiposity will be estimated from investigation of DXA scans between lumbar vertebrae L2 and L4 and L3 and L6. As the effect of abdominal adiposity on metabolic health is determined not only by total fat content but also by fat distribution[68], we will use the Siemens Sonata (Malvern, PA, USA) 1.5 Tesla magnet at Royal Prince Alfred Hospital to determine total, visceral and subcutaneous adiposity by magnetic resonance imaging (MRI), and hepatic fat content by magnetic resonance spectroscopy (MRS), which correlates closely with triglyceride analysis of liver biopsies[69]. Additionally, as evidence links thigh fat content to reduced risk of metabolic disease as recently reviewed by Associate Professor Amanda Sainsbury-Salis[35], and as reduced muscle mass predicts adverse events such as impaired gait, disability and falls[70], MRI will also be performed on the thigh to determine thigh fat content and thigh muscle diameter. Abdominal MRI studies will be performed at the level of the L4-L5 inter-vertebral disc. Mid-thigh MRI studies will be performed at the midpoint between the patella and inguinal crease. All data will be analysed

using the SliceOmatic Version 4.2 rev-1 software package (Tomovision Inc, Montreal, QC, Canada). All MRI and MRS methodology has been previously established and published by Professor Nuala Byrne. Bone mineral density will be determined by DXA at lumbar spine and hip as published in high impact journals by Associate Professor Jackie Centre[65]. As Professor Byrne has shown that weight loss without strength training significantly reduces fat free mass[62], and as there may be differences in the effect of VLED and conventional diet on fat free mass, muscle strength will be measured by dynamometry, as previously published by Associate Professor Peter Liu[71]. Moreover, a 6-minute walking test will be performed at each time point (after habituation to the test at t = -0.5 months), as a general measure of functional and cardiorespiratory fitness[72]. Additionally, fasting serum glucose, insulin, triglycerides, cholesterol and glycosylated haemoglobin will be assessed at Royal Prince Alfred Hospital. Together with waist circumference and blood pressure measurements, these measures will be used to assess metabolic syndrome stratification. Vascular health will be assessed by 1 flow-mediated dilatation (FMD), before and after inflation of a blood pressure cuff as well as acute administration of the vasodilator glyceryl trinitrate; 2 EndoPAT; 3 measurement of carotid intima-media thickness using ultrasound; and 4 pulse wave velocity, an index of vascular stiffness.

Data Analysis. All data analyses will be performed using the SPSS statistical software package version 17.0 (SPSS Inc, Chicago, IL, USA). Repeated measures ANOVA will be used to compare all subjects on VLED and conventional diet with respect to changes in parameters of body composition and metabolic or vascular health. Where a significant difference between diets is detected, posthoc analyses will subsequently be used to identify the time points that differ.

Outcomes Part 2: This rigorously controlled study, using a suite of sensitive state-of-the-art indices of body composition, metabolic and vascular health, will show for the first time whether VLED has any effects on body composition, muscle strength, metabolic and vascular health relative to effects induced by weight loss by conventional diet. For instance, although it is expected that VLED will produce greater losses of total and central adiposity relative to conventional diet up to 12 months after commencement of the diet, is this benefit maintained throughout the 36-month follow-up period, or is there a stronger rebound regain than that seen after conventional diet? Additionally, although VLED may produce greater losses in fat free mass, muscle diameter, muscle strength and bone density at early time points relative to conventional diet, does any such difference persist until 36 months? Results from this investigation will shape the direction of clinical management of obesity by demonstrating the safety or otherwise of VLED and conventional diet with respect to body composition, an important determinant of long-term metabolic health and independent living.

OVERALL OUTCOMES AND SIGNIFICANCE: Unless we can develop more effective weight loss strategies for the millions of overweight or obese people in Australia, the negative impact of obesity on human lives and the national health budget in the ensuing 50 years will be intolerable. VLEDs have proven to be effective for weight loss and safe in the short term, and with their increasing clinical use it is essential to determine their long-term efficacy and safety in relation to changes in body composition that could influence subsequent development of metabolic diseases and structural diseases such as sarcopenia and osteoporosis. Our team is well placed to deliver the outcomes of this research. With over 25 years of hospital-funded clinical experience in helping people to lose weight by VLEDs and lifestyle intervention, our expertise in

integrated assessment of energy homeostasis and appetite and our state-of-the-art methodologies for measurement of body composition and metabolism, we are in a highly competitive position to be able to fully explore the promising potential of VLEDs as an effective anti-obesity treatment.

REFERENCES FOR SECTION 1.1

1. Delbridge, E. & Proietto, J. State of the science: Very Low Energy Diet for obesity. *Asia Pac J Clin Nutr* **15 S**, 49-54 (2006).
2. Mars, M., de Graaf, C., de Groot, L.C. & Kok, F.J. Decreases in fasting leptin and insulin concentrations after acute energy restriction and subsequent compensation in food intake. *AJCN* **81**, 570-577 (2005).
3. Hukshorn, C.J., Westerterp-Plantenga, M.S. & Saris, W.H. Pegylated human recombinant leptin (PEG-OB) causes additional weight loss in severely energy-restricted, overweight men. *AJCN* **77**, 771-776 (2003).
4. Westerterp-Plantenga, M.S., Saris, W.H., Hukshorn, C.J. & Campfield, L.A. Effects of weekly administration of pegylated recombinant human OB protein on appetite profile and energy metabolism in obese men. *AJCN* **74**, 426-434 (2001).
5. Doucet, E., *et al.* Appetite after weight loss by energy restriction and a low-fat diet-exercise follow-up. *International Journal of Obesity* **24**, 906-914 (2000).
6. Pasman, W.J., Saris, W.H. & Westerterp-Plantenga, M.S. Predictors of weight maintenance. *Obesity Res* **7**, 43-50 (1999).
7. Chearskul, S., Delbridge, E., Shulkes, A., Proietto, J. & Kriketos, A. Effect of weight loss and ketosis on postprandial cholecystokinin and free fatty acid concentrations. *AJCN* **87**, 1238-46 (2008).
8. Weinsier, R.L., *et al.* Energy expenditure and free-living physical activity in black & white women: comparison before and after weight loss. *AJCN* **71**, 1138-46 (2000).
9. Martin, C.K., *et al.* Effect of calorie restriction on resting metabolic rate and spontaneous physical activity. *Obesity* **15**, 2964-2973 (2007).
10. Weinsier, R.L., *et al.* Do adaptive changes in metabolic rate favor weight regain in weight-reduced individuals? *AJCN* **72**, 1088-1094 (2000).
11. Rosenbaum, M., *et al.* Effects of weight change on plasma leptin concentrations and energy expenditure. *J Clin Endocrinol Metab* **82**, 3647-3654 (1997).
12. Leibel, R.L., Rosenbaum, M. & Hirsch, J. Changes in energy expenditure resulting from altered body weight. *New England Journal of Medicine* **332**, 621-628 (1995).
13. Goran, M.I. Energy metabolism and obesity. *Medical Clinics of North America* **84**, 347-362 (2000).
14. Hukshorn, C.J., Menheere, P.P., Westerterp-Plantenga, M.S. & Saris, W.H. The effect of pegylated human recombinant leptin (PEG-OB) on neuroendocrine adaptations to semi-starvation in overweight men. *European Journal of Endocrinology* **148**, 649-655 (2003).
15. Rosenbaum, M., Hirsch, J., Murphy, E. & Leibel, R.L. Effects of changes in body weight on carbohydrate metabolism, catecholamine excretion, and thyroid function. *AJCN* **71**, 1421-1432 (2000).
16. Wadden, T.A., Mason, G., Foster, G.D., Stunkard, A.J. & Prange, A.J. Effects of a very low calorie diet on weight, thyroid hormones & mood. *IJO* **14**, 249-58 (1990).
17. Naslund, E., *et al.* Associations of leptin, insulin resistance and thyroid function with long-term weight loss in dieting obese men. *J Int Med* **248**, 299-308 (2000).

18. Douyon, L. & Schteingart, D.E. Effect of obesity and starvation on thyroid hormone, growth hormone, and cortisol secretion. *Endocrine & Metabolism Clinics of North America* **31**, 173-189 (2002).
19. Silva, J.E. The thermogenic effect of thyroid hormone & its clinical implications. *Ann Int Med* **139**, 205-13 (2003).
20. Loeb, J.N. Metabolic changes in hypothyroidism. In: Braveman LE, Utiger RD (eds). The Thyroid. . *Lippincott: C Philadelphia, PA*, pp 1064-1071 (1991).
21. Iossa, S., *et al.* Fat balance and serum leptin concentrations in normal, hypothyroid, and hyperthyroid rats. *IJO* **25**, 417-425 (2001).
22. Freitas, F.R., *et al.* The thyroid hormone receptor beta-specific agonist GC-1 selectively affects the bone development of hypothyroid rats. *J Bone Miner Res* **20**, 294-304 (2005).
23. Johnstone, A.M., *et al.* Influence of short-term dietary weight loss on cortisol secretion and metabolism in obese men. *Eur J Endocrinol* **150**, 185-194 (2004).
24. Ho, J.T., *et al.* Moderate weight loss reduces renin and aldosterone but does not influence basal or stimulated pituitary-adrenal axis function. *Horm Metab Res* **39**, 694-699 (2007).
25. Rebuffe-Scrive, M., Walsh, U.A., McEwen, B. & Rodin, J. Effect of chronic stress and exogenous glucocorticoids on regional fat distribution and metabolism. *Physiol Behav* **52**, 583-90 (1992).
26. Dallman, M.F., *et al.* Feast and famine: critical role of glucocorticoids with insulin in daily energy flow. *Frontiers in Neuroendocrinology* **14**, 303-347 (1993).
27. Natsui, K., *et al.* High-dose glucocorticoid treatment induces rapid loss of trabecular bone mineral density and lean body mass. *Osteoporos Int* **17**, 105-108 (2006).
28. Curtis, J.R., *et al.* Population-based assessment of adverse events associated with long-term glucocorticoid use. *Arthritis & Rheumatism* **55**, 420-426 (2006).
29. Panter-Brick, C., Lotstein, D.S. & Ellison, P.T. Seasonality of reproductive function and weight loss in rural Nepali women. *Human Reproduction* **8**, 684-690 (1993).
30. Paczoska-Eliasiewicz, H.E., *et al.* Attenuation by leptin of the effects of fasting on ovarian function in hens. *Reproduction* **126**, 739-751 (2003).
31. Ahima, R.S., *et al.* Role of leptin in the neuroendocrine response to fasting. *Nature* **382**, 250-252 (1996).
32. Mah, P.M. & Wittert, G.A. Obesity and testicular function. *Mol Cell Endocrinol* **316**, 180-186.
33. Klibanski, A., Beitins, I.Z., Badger, T., Little, R. & McArthur, J.W. Reproductive function during fasting in men. *J Clin Endocrinol Metab* **53**, 258-263 (1981).
34. Kamel, H.K., Maas, D. & Duthie, E.H., Jr. Role of hormones in the pathogenesis and management of sarcopenia. *Drugs & Aging* **19**, 865-877 (2002).
35. Lovejoy, J.C. & Sainsbury, A. Sex differences in obesity and the regulation of energy homeostasis. *Obesity Reviews* **10**, 154-167 (2009).
36. Rosen, C.J., *et al.* Congenic mice with low serum IGF-I have increased body fat, reduced bone mineral density, and an altered osteoblast differentiation program. *Bone* **35**, 1046-1058 (2004).
37. Baldock, P.A., *et al.* Hypothalamic control of bone formation: distinct actions of leptin and y2 receptor pathways. *J Bone Miner Res* **20**, 1851-1857 (2005).
38. Boey, D., *et al.* Peptide YY ablation in mice leads to hyperinsulinaemia & obesity. *Diabetologia* **49**, 1360-70 (2006).

39. Baldock, P.A., *et al.* Hypothalamic regulation of cortical bone mass: opposing activity of Y2 receptor and leptin pathways. *J Bone Miner Res* **21**, 1600-1607 (2006).
40. Boey, D., *et al.* PYY transgenic mice are protected against diet-induced and genetic obesity. *Neuropeptides* **42**, 19-30 (2008).
41. Misra, M., *et al.* Elevated PYY levels in adolescent girls with anorexia nervosa. *J Clin Endocrinol Metab* **91**, 1027-33 (2006).
42. Ducy, P., *et al.* Leptin inhibits bone formation through a hypothalamic relay. *Cell* **100**, 197-207 (2000).
43. Westerterp-Plantenga, M.S., Lejeune, M.P., Nijs, I., van Ooijen, M. & Kovacs, E.M. High protein intake sustains weight maintenance after body weight loss in humans. *IJO* **28**, 57-64 (2004).
44. Vincent, R.P. & le Roux, C.W. Changes in gut hormones after bariatric surgery. *Clin Endocrinol (Oxf)* **69**, 173-179 (2008).
45. Takeda, S., *et al.* Leptin regulates bone formation via the sympathetic nervous system. *Cell* **111**, 305-317 (2002).
46. Bobbioni-Harsch, E., *et al.* Relationship between sympathetic reactivity and body weight loss in morbidly obese subjects. *IJO* **28**, 906-911 (2004).
47. Sweeney, M.E., *et al.* Severe vs moderate energy restriction with and without exercise in the treatment of obesity: efficiency of weight loss. *AJCN* **57**, 127-34 (1993).
48. Brehm, B.J., *et al.* A randomized trial comparing a very low carbohydrate diet and a calorie-restricted low fat diet on body weight and cardiovascular risk factors in healthy women. *J Clin Endocrinol Metab* **88**, 1617-23 (2003).
49. Cooper, C. & Melton, L.J., 3rd. Epidemiology of osteoporosis. *Trends Endocrinol Metab* **3**, 224-229 (1992).
50. Richman, R.M., Steinbeck, K.S. & Caterson, I.D. Severe obesity: the use of VLED or standard kilojoule restriction diets. *Med J Aust* **156**, 768-770 (1992).
51. James, W.P., *et al.* Effect of sibutramine on cardiovascular outcomes in overweight and obese subjects. *NEJM* **363**, 905-917 (2010).
52. Byrne, N.M., *et al.* Weight loss strategies for obese adults: personalized weight management program vs. standard care. *Obesity (Silver Spring)* **14**, 1777-1788 (2006).
53. O'Connor, H.T., Richman, R.M., Steinbeck, K.S. & Caterson, I.D. Dexfenfluramine treatment of obesity: a double blind trial with post trial follow up. *Int J Obes Relat Metab Disord* **19**, 181-189 (1995).
54. Bryson, J.M., *et al.* Changes in glucose and lipid metabolism following weight loss produced by a very low calorie diet in obese subjects. *Int J Obes Relat Metab Disord* **20**, 338-345 (1996).
55. McMillan-Price, J., *et al.* Comparison of 4 diets of varying glycemic load on weight loss and cardiovascular risk reduction in overweight and obese young adults: a randomized controlled trial. *Arch Intern Med* **166**, 1466-1475 (2006).
56. King, N.A., *et al.* Dual-process action of exercise on appetite control: increase in orexigenic drive but improvement in meal-induced satiety. *Am J Clin Nutr* **90**, 921-927 (2009).
57. Byrne, N.M., *et al.* Influence of distribution of lean body mass on resting metabolic rate after weight loss and weight regain: comparison of responses in white and black women. *American Journal of Clinical Nutrition* **77**, 1368-1373 (2003).
58. Colley, R.C., Byrne, N.M. & Hills, A.P. Implications of the variability in time to isotopic equilibrium in the deuterium dilution technique. *Eur J Clin Nutr* **61**, 1250-1255 (2007).

59. King, N.A., Craig, S.A., Pepper, T. & Blundell, J.E. Evaluation of the independent and combined effects of xylitol and polydextrose consumed as a snack on hunger and energy intake over 10 d. *Br J Nutr* **93**, 911-915 (2005).
60. Byrne, N.M., Hills, A.P., Hunter, G.R., Weinsier, R.L. & Schutz, Y. Metabolic equivalent: one size does not fit all. *J Appl Physiol* **99**, 1112-1119 (2005).
61. Roffey, D.M., Byrne, N.M. & Hills, A.P. Day-to-day variance in measurement of resting metabolic rate using ventilated-hood and mouthpiece & nose-clip indirect calorimetry systems. *JPEN J Parenter Enteral Nutr* **30**, 426-432 (2006).
62. Hunter, G.R., *et al.* Resistance training conserves fat-free mass and resting energy expenditure following weight loss. *Obesity (Silver Spring)* **16**, 1045-1051 (2008).
63. Sainsbury, A., *et al.* Dynorphin knockout reduces fat mass and increases weight loss during fasting in mice. *Molecular Endocrinology* **21**, 1722-1735 (2007).
64. Sainsbury, A., *et al.* Y4 receptor knockout rescues fertility in ob/ob mice. *Genes and Development* **16**, 1077-1088 (2002).
65. Bliuc, D., *et al.* Mortality risk associated with low-trauma osteoporotic fracture and subsequent fracture in men and women. *Jama* **301**, 513-521 (2009).
66. Wilson, P.W., Paffenbarger, R.S., Jr., Morris, J.N. & Havlik, R.J. Assessment methods for physical activity and physical fitness in population studies: report of a NHLBI workshop. *Am Heart J* **111**, 1177-1192 (1986).
67. Sallis, J.F., *et al.* Physical activity assessment methodology in the Five-City Project. *Am J Epidemiol* **121**, 91-106 (1985).
68. Gan, S.K., *et al.* Insulin action, regional fat, and myocyte lipid: altered relationships with increased adiposity. *Obes Res* **11**, 1295-1305 (2003).
69. Johnson, N.A., *et al.* Noninvasive assessment of hepatic lipid composition: Advancing understanding and management of fatty liver disorders. *Hepatology* **47**, 1513-1523 (2008).
70. Walsh, M.C., Hunter, G.R. & Livingstone, M.B. Sarcopenia in premenopausal and postmenopausal women with osteopenia, osteoporosis and normal bone mineral density. *Osteoporos Int* **17**, 61-67 (2006).
71. Fennell, C., *et al.* Randomized cross-over clinical trial of injectable vs. implantable depot testosterone for maintenance of testosterone replacement therapy in androgen deficient men. *Clin Endocrinol (Oxf)* (2009).
72. Hulens, M., Vansant, G., Claessens, A.L., Lysens, R. & Muls, E. Predictors of 6-minute walk test results in lean, obese and morbidly obese women. *Scand J Med Sci Sports* **13**, 98-105 (2003).

1.2 Final protocol (link to publication)

Our final protocol is published open access in the following manuscript (digital object identifier: 10.3390/healthcare6030085).

Seimon RV, Gibson AA, Harper C, Keating SE, Johnson NA, da Luz FQ, Fernando HA, Skilton MR, Markovic TP, Caterson ID, Hay P, Byrne NM, Sainsbury A. Rationale and protocol for a randomised controlled trial comparing fast versus slow weight loss in postmenopausal women with obesity – the TEMPO Diet Trial. Healthcare (Basel) 2018;6.

1.3 Summary of changes from original protocol

1.3.1 Changes made before recruitment of first participant

The first participant was recruited into the TEMPO Diet Trial on 24 March 2014, as listed on the Australian New Zealand Clinical Trials Registry (Number 12612000651886). As such, all of the following changes (made on 29 November 2013) occurred before recruitment of the first participant.

- Title was changed to The TEMPO Diet Trial: **T**ype of **E**nergy **M**anipulation for **P**romoting optimum metabolic health and body composition in **O**besity. This title was developed as part of our recruitment strategy.

- Using the very low energy diet (VLED) for a period of 4 months, instead of a minimum of 3 months and a maximum of 5 months. This change was made to reduce variability in the results. Patients at the Metabolism & Obesity Services at Royal Prince Alfred Hospital routinely use VLEDs for periods of 4 months.

- Three additional inclusion criteria and a screening step were added. For safety, participants must not have any contraindications for following a VLED, and they must not have donated whole blood in the 3 months preceding the trial. As this trial is labour intensive for both investigators and participants, we also needed to ensure that potential participants would be capable of completing activities required for this trial, such as keeping food, activity and sleep diaries at home, wearing accelerometers and temperature trackers for 7 days at a time, and keeping appointments with our team. Thus, after signing the consent form, potential participants would be required to complete these screening activities prior to being randomised for the trial.

- One exclusion criterion was slightly modified. Instead of ‘no metal in the body’, this was changed to ‘no loose metal in the body’. Unlike loose metal such as pacemakers or bullets, metal that is fixed, such as titanium fastened to bones in joint replacements, is not a contraindication for the magnetic resonance imaging and magnetic resonance spectroscopy to be used in this trial.

- The provider of the VLED meal replacement formula was changed from Nestlé (Optifast®) to Prima Health Solutions (KicStart™). Both Optifast® and KicStart™ VLEDs are routinely used in the Metabolism & Obesity Services at Royal Prince Alfred Hospital, because an independent study has shown both to be the most nutritionally complete VLED products available. Prima Health Solutions was able to offer their product, KicStart™, at a lower cost than Nestlé.

- Fasting urinary and whole blood ketone concentrations would be measured, instead of fasting serum ketone levels as in the original protocol, because measurements of ketones in urine and whole blood are more common than serum measurements.

- The protocol for the appetite test was changed to reflect current literature. Instead of consuming one meal replacement formula as a liquid meal, participants would consume a meal that more closely resembled a normal breakfast (toast, margarine, eggs, orange juice). This was necessary to more accurately assess changes in appetite over the course of the trial. In addition, 2 mL of blood would be taken from a venous cannula at 7 time points during the appetite test. These samples would be used to assess plasma concentrations of gut-derived appetite-regulating hormones, notably ghrelin, peptide YY and leptin, in order to provide greater depth of understanding of any associated changes in appetite.

- Pedometers would be used to assess activity, in addition to the accelerometers approved for use in the original protocol. The pedometer would be given to each participant to keep, to help promote regular and ongoing physical activity.

- Body temperature would be assessed by continuously measuring peripheral skin temperature on the upper arm for up to 7 days at a time.

- The list of bone turnover markers to be measured was refined. This change would enable the most cost-effective analysis of bone turnover markers.

- The list of questionnaires to be used in this trial was expanded. This change was implemented following discussion with our collaborators, with the aim of maximising information obtained from this trial. It would enable us to determine whether fast versus slow weight loss has differential effects on eating behaviours such as bingeing or purging, as well as general mental health, mindfulness and personality.

- Vascular function and structure tests were modified to reduce costs (including staffing costs) and participant burden. Glyceryl trinitrate (GTN) would no longer be used during measurement of flow-mediated dilatation (FMD). EndoPAT or pulse wave velocity tests of vascular health would no longer be used.

- One additional dual energy X-ray absorptiometry (DXA) scan was added to the protocol. Its purpose was to check for osteoporosis, an exclusion criterion for our trial. It would occur during screening, for participants who have not had a clinical DXA scan to assess bone density within the past 2 years, or for participants who have had such a scan within the past 2 years but whose result was close to the definition of osteoporosis. This additional DXA scan would not be used for data analysis.

- Sympathetic reactivity (using doppler fluximetry) was not included in the final protocol, as we did not have access to equipment to measure this.

- The 6-minute walking test was removed from the final protocol, as we did not have access to a non-slip under-cover venue in which to conduct this test uniformly in varying weather conditions.

1.3.2 Changes made after recruitment of first participant

With the first participant being recruited on 24 March 2014 (Australian New Zealand Clinical Trials Registry Number 12612000651886), the following change (implemented on 9 July 2014) was made after baseline testing of the first 13 participants.

- The Loss of Control Over Eating Scale (LOCES) was added after discussion and agreement with collaborators that this measure would provide additional insights into eating behaviour (including eating disorder behaviours) that was not available from the other questionnaires being used in the trial.

2 Statistical analysis plan

2.1 Original statistical analysis plan

Our original statistical analysis plan is in our original protocol, under the headings of "Database maintenance and data analysis" and "Data Analysis".

2.2 Final statistical analysis plan

A summary of our final statistical analysis plan is included in the current manuscript, under the heading of "Statistical Analysis". A detailed version of this is below.

For our primary outcome of whole-body lean mass at 12 months after intervention commencement, we calculated that a target sample size of 100 participants would provide a power of 90% at a two-sided alpha level of 5%, allowing for up to 20% attrition as seen in previously-published weight loss interventions.[1-3] Notably, attrition at 12 months in our current trial was 16% (to be detailed in the Results section), which fell within the attrition prediction used in our sample size calculation. Specifically, to detect a 5% difference in whole-body lean mass (e.g. 50.3 kg versus 53.0 kg, and assuming a variance of 5 kg), the effect size is 0.52, so at 90% power and P<0.05, a sample size of 39 participants per group would be necessary, therefore with 20% attrition we would need 50 participants in each group. Further, based on studies in participants with obesity that elicited comparable weight losses to that anticipated from the SEVERE intervention in this trial, i.e. 12-20% of initial body weight over 12 months, the effect size to detect differences between interventions in the change in bone mineral density are large (>0.8), indicating that a sample of 20 per group would provide 90% power to detect significant (P<0.05) differences between interventions.

Statistical analyses were performed using SPSS Statistics Version 24 for Windows (IBM Corporation, New York, NY, USA). Statistical significance was accepted as P<0.05. Fisher's exact tests were used to compare categoric demographic characteristics (age, ethnicity) and attrition between groups (i.e. SEVERE versus MODERATE). To compare continuous variables between groups at baseline (0 months), Mann-Whitney tests were used. All continuous variables were assessed for normality before analysis using histograms and P-P-plots. Where data were not normally distributed, a natural log-transformation was applied to obtain a normal distribution, although means and confidence intervals are reported on untransformed data.

To compare longitudinal changes between groups, intention-to-treat analysis was performed using data from all participants originally randomized, using random effects linear mixed models. Mixed model analyses were used instead of standard repeated measures analysis of variance because of the likelihood that there would be dropouts and missed visits that preclude the use of the classical approach.[4] Therefore, this model allowed for participants to have partial missing data and still be included without imputation. Intervention group and time (0, 4, 6, and 12 months for all parameters except for waist circumference, hip circumferences, and waist to hip ratio, which were assessed at all of these points plus at 0.25 and 1 months), were included as fixed effects, and participant was included as a random effect and included an intercept. Nominal

time was used, although measurements at 0 and 0.25 months were always done exactly 1 week apart, measurements at 1 month were done within ±2 days from the exact date, measurements at 4 and 6 months were done within ±1 week from the exact date, and measurements at 12 months were done within ±2 weeks from the exact date. For all outcomes, baseline values of the relevant variable were added as a covariate in the analysis because when, by chance, there happens to be imbalance in the baseline covariate between groups, even when the two treatment groups are well balanced, adjusting for the covariates will (for linear models) give a more precise estimate of the treatment effect.

As the degree of physical activity is known to influence measures of body composition, physical activity (expressed in metabolic equivalents of task, or METs) was added as a covariate, as measured at each of the timepoints. Maximum-likelihood estimation was used, and an unstructured covariance matrix was specified to account for the correlation between repeated measures over time by allowing the intercept for individuals to vary randomly. When the overall P value for the interaction between group and time was less than 0.05, Bonferroni adjustments were used to correct for multiple comparisons between groups at each time point. As a secondary analysis, the model was further adjusted for weight at each time point, because lighter individuals are known to have lower whole-body lean mass and BMD than heavier individuals.[5,6] This was to determine if there was an effect of the interventions beyond that which would be expected due to weight loss.

Within-group changes were analysed with repeated measures linear mixed models. Intervention group and time were included in the model as fixed effects, and time as a repeated measure, with physical activity (METs) added as a covariate. Maximum-likelihood estimation was used, and an unstructured covariance matrix was specified. When the overall P value for the interaction between group and time was less than 0.05, Bonferroni adjustments were used to correct for multiple comparisons between time points and baseline (0 months) within each group.

2.3 Summary of changes from original statistical analysis plan

The final statistical analysis plan provides details that were not defined in our original statistical analysis plan.

Whereas our original statistical analysis plan included the use of repeated measures ANCOVA or ANOVA, our final statistical analysis plan used mixed models, in order to allow for missing data.[7]

References for Sections 2.2 and 2.3

1. Bryson JM, King SE, Burns CM, Baur LA, Swaraj S, Caterson ID. Changes in glucose and lipid metabolism following weight loss produced by a very low calorie diet in obese subjects. *International journal of obesity and related metabolic disorders : journal of the International Association for the Study of Obesity*. 1996;20(4):338-345.

2. Byrne NM, Meerkin JD, Laukkanen R, Ross R, Fogelholm M, Hills AP. Weight loss strategies for obese adults: personalized weight management program vs. standard care. *Obesity (Silver Spring).* 2006;14(10):1777-1788.
3. McMillan-Price J, Petocz P, Atkinson F, et al. Comparison of 4 diets of varying glycemic load on weight loss and cardiovascular risk reduction in overweight and obese young adults: a randomized controlled trial. *Archives of internal medicine.* 2006;166(14):1466-1475.
4. Gadbury GL, Coffey CS, Allison DB. Modern statistical methods for handling missing repeated measurements in obesity trial data: beyond LOCF. *Obesity reviews : an official journal of the International Association for the Study of Obesity*. 2003;4(3):175-184.
5. Salamat MR, Salamat AH, Janghorbani M. Association between Obesity and Bone Mineral Density by Gender and Menopausal Status. *Endocrinol Metab (Seoul).* 2016;31(4):547-558.
6. Bales CW, Buhr G. Is obesity bad for older persons? A systematic review of the pros and cons of weight reduction in later life. *Journal of the American Medical Directors Association.* 2008;9(5):302-312.
7. Gadbury GL, Coffey CS, Allison DB. Modern statistical methods for handling missing repeated measurements in obesity trial data: beyond LOCF. Obesity reviews : an official journal of the International Association for the Study of Obesity 2003;4:175-84.
