# Supplementary material for: Effect of Weight Loss via Severe vs Moderate Energy Restriction on Lean Mass and Body Composition Among Postmenopausal Women With Obesity: The TEMPO Diet Randomized Clinical Trial
Source: JAMA Netw Open. 2019 Oct 30;2(10):e1913733. doi: 10.1001/jamanetworkopen.2019.13733 (PMC6824325; doi:10.1001/jamanetworkopen.2019.13733)
Supplement: Supplement 2. — eFigure. Effect of Severe vs Moderate Energy Restriction on Body Composition in Postmenopausal Women With Obesity [file jamanetwopen-2-e1913733-s002.pdf]

Supplementary Online Content

Seimon RV, Wild-Taylor AL, Keating SE, et al. Effect of weight loss via severe vs moderate energy restriction on lean mass and body composition among postmenopausal women with obesity: the TEMPO Diet randomized clinical trial. *JAMA Netw Open*. 2019;2(10):e1913733. doi:10.1001/jamanetworkopen.2019.13733

eFigure. Effect of Severe vs Moderate Energy Restriction on Body Composition in Postmenopausal Women With Obesity

This supplementary material has been provided by the authors to give readers additional information about their work.

© 2019 Seimon RV et al. *JAMA Network Open.*

eFigure. Effect of Severe vs Moderate Energy Restriction on Body Composition in Postmenopausal Women with Obesity

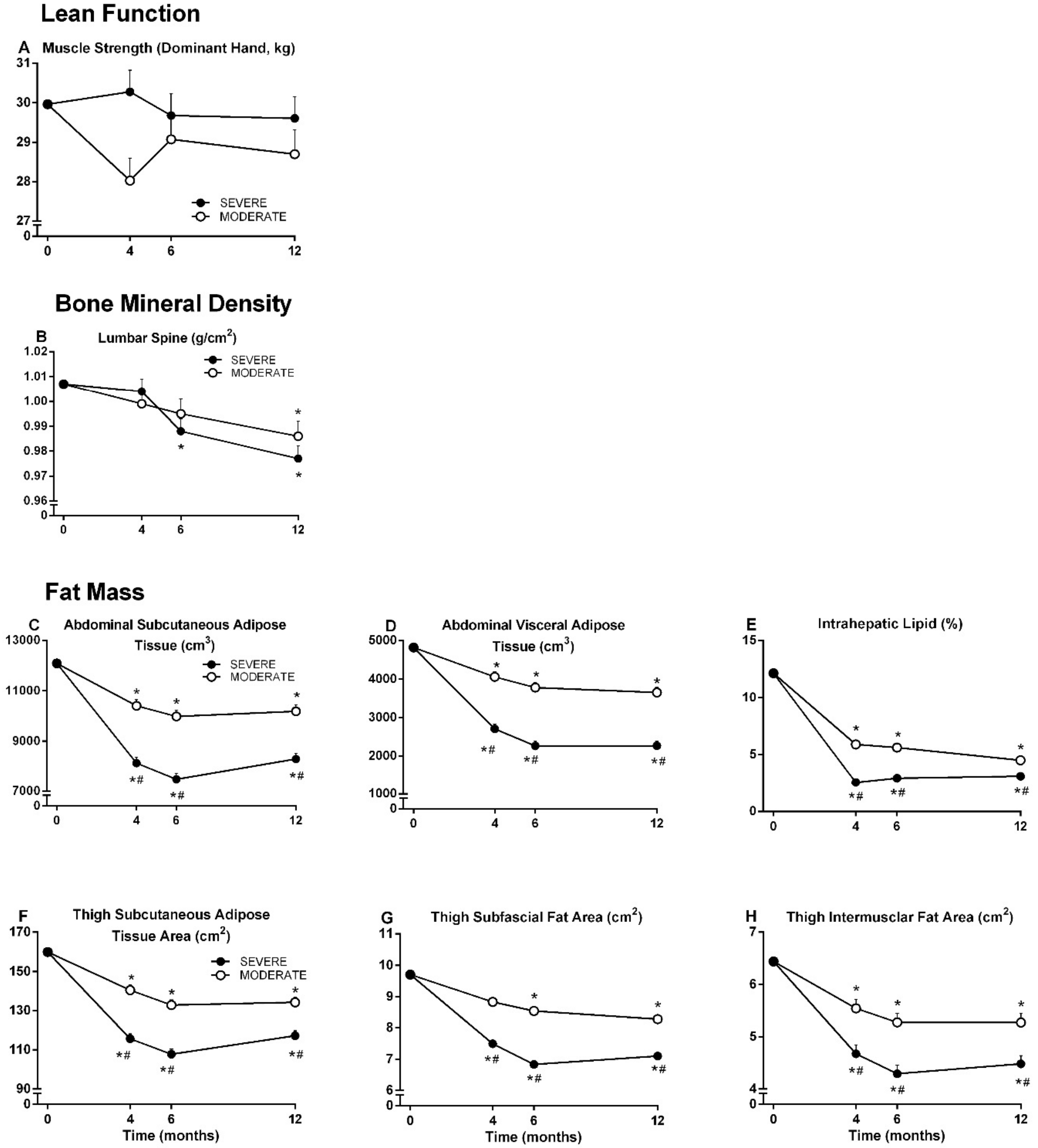

Mean of lean function: Muscle strength of the dominant hand (A); bone mineral density: lumbar spine (B); fat mass: abdominal subcutaneous adipose tissue (C), abdominal visceral adipose tissue (D), intrahepatic lipid (E), thigh subcutaneous adipose tissue area (F), thigh subfascial fat area (G), and thigh intramuscular fat area (H), during the 12-month severe or moderate intervention. Data presented as estimated marginal means (i.e. group means after controlling for

© 2019 Seimon RV et al. *JAMA Network Open.*

covariate/s); where data were not normally distributed (i.e. intrahepatic lipid and subfascial thigh fat area) data presented as geometric means; baseline values presented on the graph were the covariates in the statistical analysis model; T bars indicate standard errors of the means. * indicates P<0.05 versus baseline value (0 months) within group. # indicates P<0.05 versus the moderate group at that point.

© 2019 Seimon RV et al. *JAMA Network Open.*
