# Supplementary material for: Effect of Weight Loss via Severe vs Moderate Energy Restriction on Lean Mass and Body Composition Among Postmenopausal Women With Obesity: The TEMPO Diet Randomized Clinical Trial
Source: JAMA Netw Open. 2019 Oct 30;2(10):e1913733. doi: 10.1001/jamanetworkopen.2019.13733 (PMC6824325; doi:10.1001/jamanetworkopen.2019.13733)
Supplement: Supplement 3. — Data Sharing Statement [file jamanetwopen-2-e1913733-s003.pdf]

Data Sharing Statement

Seimon. Effect of Weight Loss via Severe vs Moderate Energy Restriction on Lean Mass and Body Composition Among Postmenopausal Women With Obesity. *JAMA Netw Open*. Published October 30, 2019. 10.1001/jamanetworkopen.2019.13733

Data
Data available: No
